# Bidirectional Exchange of Biogenic Volatile Organic Compounds in Subarctic Heath Mesocosms During Autumn Climate Scenarios

**DOI:** 10.1029/2021JG006688

**Published:** 2022-06-20

**Authors:** Nanna S. Baggesen, Cleo L. Davie‐Martin, Roger Seco, Thomas Holst, Riikka Rinnan

**Affiliations:** ^1^ Terrestrial Ecology Section, Department of Biology University of Copenhagen Copenhagen Ø Denmark; ^2^ Center for Permafrost (CENPERM) University of Copenhagen Copenhagen K Denmark; ^3^ Institute of Environmental Assessment and Water Research (IDAEA‐CSIC) Barcelona Spain; ^4^ Department of Physical Geography and Ecosystem Science Lund University Lund Sweden

**Keywords:** arctic, autumn, global change, flooding, volatile organic compound, ecosystem‐atmosphere interactions

## Abstract

Biogenic volatile organic compound (BVOC) flux dynamics during the subarctic autumn are largely unexplored and have been considered insignificant due to the relatively low biological activity expected during autumn. Here, we exposed subarctic heath ecosystems to predicted future autumn climate scenarios (ambient, warming, and colder, dark conditions), changes in light availability, and flooding, to mimic the more extreme rainfall or snowmelt events expected in the future. We used climate chambers to measure the net ecosystem fluxes and bidirectional exchange of BVOCs from intact heath mesocosms using a dynamic enclosure technique coupled to a proton‐transfer‐reaction time‐of‐flight mass spectrometer (PTR–ToF–MS). We focused on six BVOCs (methanol, acetic acid, acetaldehyde, acetone, isoprene, and monoterpenes) that were among the most dominant and that were previously identified in arctic tundra ecosystems. Warming increased ecosystem respiration and resulted in either net BVOC release or increased uptake compared to the ambient scenario. None of the targeted BVOCs showed net release in the cold and dark scenario. Acetic acid exhibited significantly lower net uptake in the cold and dark scenario than in the ambient scenario, which suggests reduced microbial activity. Flooding was characterized by net uptake of the targeted BVOCs and overruled any temperature effects conferred by the climate scenarios. Monoterpenes were mainly taken up by the mesocosms and their fluxes were not affected by the climate scenarios or flooding. This study shows that although autumn BVOC fluxes on a subarctic heath are generally low, changes in future climate may strongly modify them.

## Key Points

- Biogenic volatile organic compound (BVOC) fluxes in the autumn ecosystem are mainly derived from soil microbial activity
- Flooding results in negative net BVOC exchange, mainly due to BVOC deposition
- Autumnal warming enhances vegetation growth and soil respiration, resulting more often in net BVOC emissions than under colder conditions

## 1 Introduction

Most arctic field research into biogenic volatile organic compounds (BVOCs) ends in late summer, before the first snow, and is first initiated again after snowmelt, in early summer (Baggesen et al., 2021; Faubert et al., 2010; Tiiva et al., 2008; Valolahti et al., 2015). BVOC dynamics during the long autumn and winter periods are largely unexplored because biological productivity is relatively low and plants are inactive and unproductive. Although BVOC fluxes are expected to be low during autumn, their dynamics during these periods, particularly during extreme weather events and with shifting future climate patterns, are important to gain a full picture of the yearly variation.

BVOCs are mainly emitted from green plants and flowers (Peñuelas et al., 2014), plant litter (Greenberg et al., 2012; Svendsen et al., 2018), and soils (Aaltonen et al., 2011; Tang et al., 2019), whereas uptake is highest in soils (Albers et al., 2018; Rinnan & Albers, 2020). Thus, the highest BVOC emissions occur during peak summer, when leaf biomass has its maximum (Baggesen et al., 2021; Tang et al., 2018), and the highest BVOC uptake by the ecosystem, relative to emissions, is presumed to occur outside the growing season (Seco et al., 2020).

The arctic autumn is characterized by shifting weather and huge variations in temperature, ranging from clear skies and two‐digit positive Celsius degrees to heavy rain or snow‐cover and below freezing temperatures. A study by Mastepanov et al. (2008) showed that during the autumn freeze‐in of arctic soils, bursts of methane were released equivalent to the amounts emitted during summer, which could indicate that other gaseous compounds might also be released in larger quantities during the autumn. Furthermore, it has previously been demonstrated that rain events might also lead to BVOC emission bursts, due to rapid soil microbial responses to the changed moisture availability (Greenberg et al., 2012; Rossabi et al., 2018). Thus, weather fluctuations are important when looking at yearly BVOC flux dynamics. As the climate changes, weather fluctuations may result in extreme events (Hansen et al., 2014), such as heavy snowfall, causing snowmelt delays in spring (Semenchuk et al., 2016), and heavy rain, leading to flooding and ice cover when temperatures go below freezing (Hansen et al., 2014). Formation of ice on the terrestrial ecosystem has a negative effect on both soil and vegetation processes causing damage to vascular plants (Bjerke, 2011) and reduced survival of soil microbes (Coulson et al., 2000), while a snowpack can provide an insulating layer that decreases temperature fluctuations and sustains soil microbial activity (Schimel et al., 2004). In the absence of ice layers within the snowpack, active ecosystem‐atmosphere exchange can take place through snow (Aaltonen et al., 2012; Meredith et al., 2017).

Flooding causes anaerobic conditions in the soil and typically initiates fermentation processes, resulting in reduced ecosystem activity and the production of different BVOCs (e.g., ethanol, acetaldehyde, and acetic acid) compared to oxidative processes (Fall 2003; Faubert et al., 2011; Rinnan et al., 2014; Seco et al., 2007). Furthermore, flooding may delay BVOC emissions from roots and soils, due to slower gas diffusion in water compared to air (Rottenberger et al., 2008), and reduced microbial uptake under anaerobic conditions (Faubert et al., 2010). Water‐soluble compounds might also dissolve in water in greater amounts when ecosystems are flooded. BVOC emissions are expected to arise almost exclusively from above‐ground plants in flooded ecosystems, because the high water table limits the contributions from soils and below‐ground microbes, and may therefore, result in changes to the BVOC compositions leaving and entering the ecosystem (Faubert et al., 2011; Tiiva et al., 2009).

Climate warming in the Arctic increases the fluxes of temperature‐sensitive BVOCs from the ecosystem during the growing season (Baggesen et al., 2021; Faubert et al., 2010; Lindwall et al., 2016; Tiiva et al., 2008; Valolahti et al., 2015), whereas autumnal warming could be hypothesized to only have a minor effect on BVOC emissions, due to the natural decrease in green biomass during the autumn (Anderson et al., 2016) and hence, decrease in vegetation‐derived BVOC production. Although vegetation is less active at the onset of autumn, there may still be a clear vegetation‐dominated emission (Rinnan et al., 2013). Two studies from the Mediterranean showed that the effect of warming on ecosystems is highly dependent on the hydrological conditions of the specific site (Asensio et al., 2007, 2008). Yan et al. (2021) showed that year‐round warming only delayed foliar senescence and increased growth (i.e., biomass) in the autumn, in years with high precipitation in cold, semi‐humid climates, a scenario that falls within climate projections (Bintanja et al., 2020). Future autumn temperatures will increase as a result of global warming (Post et al., 2019) and combined with sufficient moisture conditions, will accelerate the activity of the soil microbial community and organic matter decomposition, which may increase soil BVOC emissions (Aaltonen et al., 2011). The increases in plant biomass and soil decomposition rates might lead one to expect that autumn BVOC fluxes are strongly affected and may be of increasing importance, which highlights the relevance of studying autumnal BVOC fluxes.

Here, we conducted a laboratory experiment to investigate the dynamics of the bidirectional BVOC exchange in subarctic tundra heath mesocosms (i.e., intact blocks of tundra containing plants, soil, and their associated microbes), when exposed to different autumn weather scenarios. We sampled net BVOC ecosystem fluxes to estimate the effect of warming, darkness, and cooling, as well their interaction with flooding during the autumn. We hypothesized that, (a) experimental warming under natural autumn light conditions would increase the net release of light‐dependent BVOCs (Baggesen et al., 2021), (b) the colder and dark climate scenario (cooling_dark) would decrease bidirectional BVOC fluxes (i.e., the magnitude of net uptake and net release) and change the compound composition, due to reduced light availability lowering light‐dependent release and colder temperatures reducing microbial metabolism (Lindwall et al., 2015), (c) flooding would halt aerobic decomposition processes and shift soil communities to fermentation, which would change the compound composition of the BVOC fluxes (Faubert et al., 2011), and (d) there would be a difference in BVOC composition and fluxes between the beginning and end of the autumn period, due to phenology changes in the vegetation (Baggesen et al., 2021).

## 2 Materials and Methods

### 2.1 Site Description

Twenty mesocosms were collected from a slightly sloped, subarctic heath in Abisko, northern Sweden (68°210'N, 18°490'E, 385 m a.s.l.) in July 2018. The collection site was close to an experimental field where soil characteristics (Baggesen et al., 2021; Lett & Michelsen, 2014) and BVOC fluxes (Baggesen et al., 2021; Faubert et al., 2010; Valolahti et al., 2015) have previously been reported. Vegetation in the area is mixed, low (canopy height 5–15 cm) tundra heath vegetation composed of evergreen and deciduous dwarf shrubs, graminoids, and a few herb species, as well as mosses. For further site description, see Baggesen et al. (2021). Mesocosm collection was performed by cutting and digging out an 18 × 18 × 11 cm (length × width × height) square of soil including the above and below ground organic material and intact vegetation and transferring those samples into polypropylene containers (Ghirardo et al., 2020). As the vegetation composition is naturally variable, the mesocosms can be considered to represent natural heterogenic heath tundra. The mesocosms were transported to Copenhagen, Denmark within a week, where for practical purposes, they were stored outside from July 2018 until March 2019. From October 2018 to March 2019, the mesocosms were sheltered under a roof cover to decrease solar radiation and protect them from frost. Our goal was to mimic autumn conditions and the temperatures in Copenhagen, Denmark were expected to be similar to those the plants would experience under a deep insulating snow layer. However, the mesocosms did experience low levels of longer daylight periods and as such, the storage conditions may have affected the plant phenology.

### 2.2 Experimental Setup

**Figure 1:**
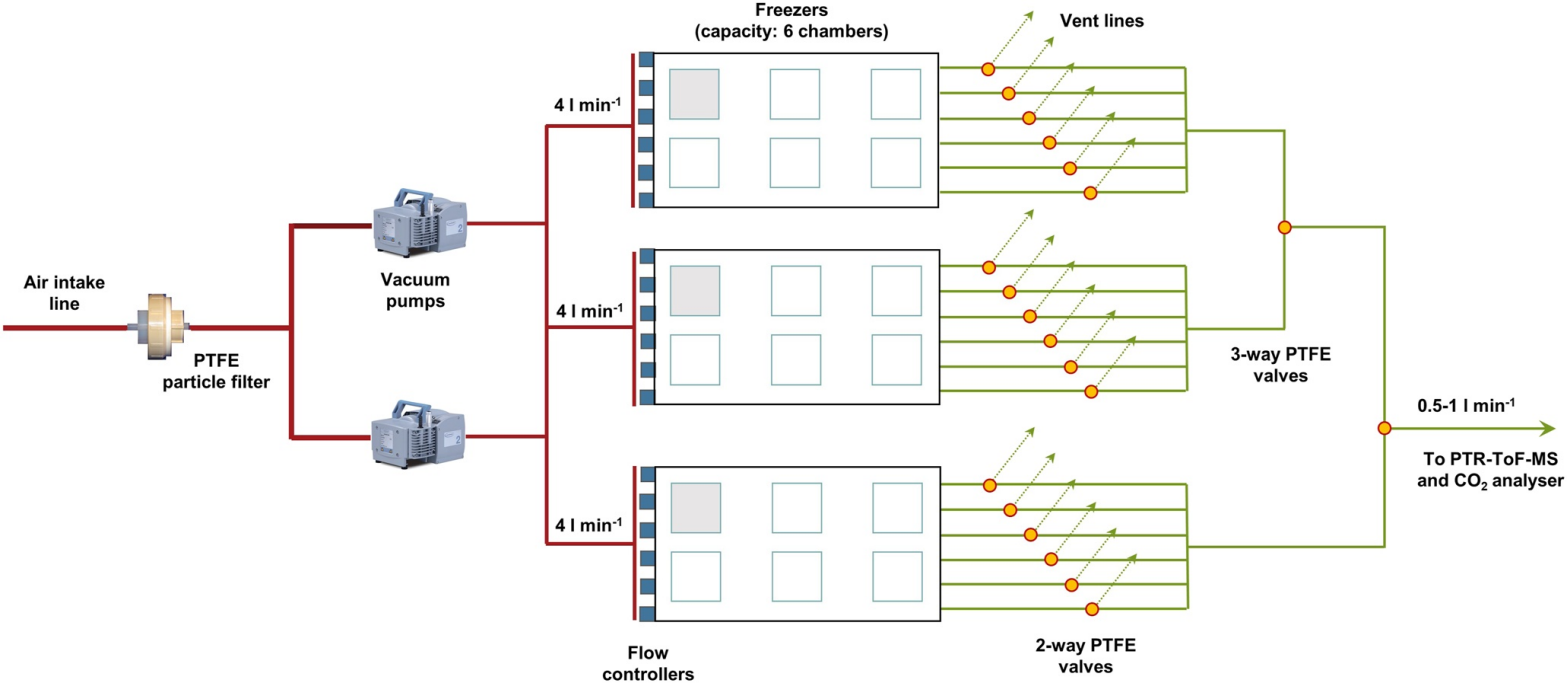
Schematic for freezer set‐up. Red and green lines represent perfluoralkoxy (PFA) tubing. Gray chambers indicate blanks. For individual freezer layout see Figure S1 in Supporting Information S1.

We used a set of custom‐built climate chambers constructed from three regular commercial deep freezers (Whirlpool, WHM3911, Michigan, USA) whose lids were replaced by Plexiglas sheets with adjustable plant growth light sources (Valoya C65 with NS12 spectrum, Helsinki, Finland) to mimic natural sun light. A fan was installed in the bottom of each freezer to circulate the air and provide a more uniform temperature distribution (Figure S1 in Supporting Information S1). Daytime PAR (photosynthetically active radiation) intensity was set to the same level for all freezers (200 μmol m^−2^ s^−1^), which was the expected intensity for Abisko in October (Table S1 in Supporting Information S1). Six transparent polycarbonate chambers (wall thickness 3 mm, 21 × 21 × 30 cm length × width × height, volume 13.23 L) with removable lids were installed in each freezer, with ports for independent supply air and sample outflow air lines. Each of the 18 chambers contained a shaded temperature data logger (Hygrochron DS 1923‐F5 iButton, Maxim Integrated Products Inc., CA, USA) to record air temperature in the chamber headspace every 5 minutes. The mesocosms were randomly split into four groups (*n* = 5 each) and 15 of those mesocosms were placed inside the chambers. Each freezer contained five chambers with a mesocosm inside and one empty chamber, used as a blank (Figure 1). The remaining five mesocosms were used for initial soil analyses.

The air supply to the chambers was pulled from the roof of a six‐story building through a ventilation shaft, by two diaphragm vacuum pumps (Vacuubrand ME 2 NT, Wertheim, Germany). Supply air from the roof provided lower and more stable background concentrations of VOCs compared to intake air nearer the ground with VOC point sources from vegetation and anthropogenic activities (e.g., idling traffic, cigarette smoke). Supply air was filtered through a Savillex 90 mm PTFE membrane (Eden Prairie, Minneapolis) to remove dust particulates. VOCs were not removed from the incoming air because using VOC‐free air impedes the potential bidirectional exchange of VOCs. The final two meters of the supply air tubing (3/8” O.D. perfluoralkoxy, PFA, tubing, Swagelok, Esbjerg, Denmark) was pre‐cooled in the freezer to avoid unwanted heating in the chambers (Figure S1 in Supporting Information S1) before the airflow was split into individual supply lines (1/4” O.D. PFA tubing; flow rate of ∼4 L min^−1^) to each chamber to apply the dynamic enclosure technique (Figure 1). All supply lines to the dynamic enclosures inside the freezers were thermally insulated to prevent condensation from freezing, which could block the airflow. Data from periods when the airflow was blocked by ice formation (mainly during the night) were excluded (≈56% excluded).

Our experimental design comprised of three climate scenarios and each freezer was programmed with different temperature and light regimes depending on the climate scenario. In the “ambient” climate scenario, the freezer experienced regular October conditions for Abisko, with temperatures around 0°C during the night and up to 15°C during the day, with 6 hr of daylight (i.e., when the lights were on). The second freezer experienced a colder and darker autumn (climate scenario termed “cooling_dark”), where the temperatures were 0°C and 5°C during the night and day, respectively. In the cooling_dark scenario, lights remained off during the day and night, except during 6‐hr periods of daylight on the 20 and 25 March, and the 3 and 8 April, used to mimic short warming events (i.e., 2 days out of each 12‐day experiment). The third freezer experienced a “warming” climate scenario with temperatures ranging between 10°C during the night and up to 20°C during the day, with 6 hr of daylight (Figure S2 in Supporting Information S1). Our study was divided into two 12‐day experimental periods termed “pre‐flooding” (March 18–29, 2019) and “flooding” (April 1–12, 2019). In the 3‐day period between these experiments, mesocosms were removed from the freezers and stored at 20°C for practical reasons. During the subsequent flooding experiment, demineralized water was added to the mesocosms so that the ground water table was level with the soil surface (Table S2 in Supporting Information S1). Mesocosms were exposed to the same climate scenario in both the pre‐flooding and flooding experiments. During the flooding experiment, soils in mesocosms from the ambient and cooling_dark climate scenarios froze below a depth of 2–5 cm.

Throughout the experiment, mesocosms were rotated every third day within each freezer to ensure that all mesocosms inside that particular freezer experienced the same light and temperature conditions.

### 2.3 Soil Analyses

**Figure 2:**
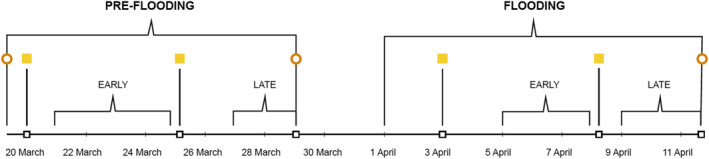
Experimental timeline. The study ran for 2× 12‐day experiments (Pre‐flooding and Flooding). During the first 12 days, three different temperature regimes (climate scenarios) ran in parallel: Ambient, Cooling_dark, and Warming. During the second 12 days, the same three climate scenarios were employed, but this time, the mesocosms were flooded. Each of the 12‐day experiments were split into an Early and a Late period covering 2–4 days. Yellow squares indicate days where the lights were periodically on (for 6 hr) in the cooling_dark climate scenario, and brown circles indicate soil sampling days. A full timeline of background CO_2_ and BVOC mixing ratios and the corresponding measured net fluxes for net ecosystem exchange (NEE) of CO_2_ and each of the six focus BVOCs are provided in Figures S4–S17 in Supporting Information S1.

A soil corer (2.5 cm diameter) was used to retrieve triplicate soil cores to 5 cm depth from each mesocosm. The cores were divided into two depths, 0–2 cm and 2–5 cm, and the three subsamples were pooled together as one sample per depth and mesocosm. Soil samples were collected three times: (a) before the experimental start on five randomly selected mesocosms that were not used in the experiment (19 March), (b) after the pre‐flooding experimental period in all mesocosms (29 March), and (c) after the flooding period (12 April, Figure 2).

To analyze for pH, 2.5 g of soil was extracted with 12.5 ml of distilled water, mixed for 1 hr on an orbital shaker and centrifuged for 10 min, before measurement with a MeterLab 240 electronic pH‐meter (Mettler Toledo, Denmark). To analyze for carbon and nutrients, 5 g of soil was extracted in 25 ml of distilled water and processed the same way as for the pH analysis. The extract was then filtered through a Whatman GF/D glass microfiber filter (Darmstadt, Germany). Total dissolved organic carbon was analyzed on a Shimadzu TOC5000A analyzer (Shimadzu, Kyoto, Japan). Total dissolved nitrogen, phosphate (PO_4_^3−^), ammonium (NH_4_^+^), and nitrate (NO_3_^−^) were measured with a flow injection analyzer (5000 FIASTAR, Höganäs, Sweden).

A fraction of the soil was used to determine gravimetric soil water content by weighing the soil before and after it was dried at 60°C.

### 2.4 Vegetation Analysis

During the mesocosm rotations, that is, every 3 days, greenness index was measured using the Canopeo application (Oklahoma State University, 2015), which measures the percent canopy cover of live green vegetation. For comparability, greenness index measurements were always performed under the same light conditions.

Aboveground plant biomass was determined immediately after the experiment ended. The mesocosms were harvested leaving only bare soil, sorted by species, and weighed after oven drying (Table S3 in Supporting Information S1).

### 2.5 BVOC and CO_2_ Mixing Ratio Measurements

Trace gas fluxes were measured using a dynamic enclosure technique at the mesocosm level. A CR1000 data logger coupled with an SDM‐CD8S controller (both Campbell Scientific, Logan, Utah) was used to control the switching times of an array of two‐ and three‐way PTFE solenoid valves (Cole‐Parmer, Cambridgeshire, UK), which allowed each mesocosm to be sampled once per hour (Figure 1). The five mesocosms in one freezer were measured individually, in sequence, for 2.5 min each, and all separated by 1.5 min blank measurements from the empty chamber in the same freezer. Measurements from one freezer were completed (a 20‐min program) before switching to the next freezer (Table S4 in Supporting Information S1).

BVOC mixing ratios were measured using a PTR‐TOF‐1000 ultra (Ionicon Analytik, Innsbruck, Austria) at 5‐s resolution. Conditions of 2.3 mbar, 60°C, and 550 V were used in the drift tube.

Measurements of net ecosystem exchange (NEE) of CO_2_, taken from a T‐piece split parallel to the PTR–ToF–MS, were performed by a LI840A CO_2_/H_2_O infrared gas analyzer (LI‐COR Biosciences, Lincoln NE, USA) (Figure 1). A pocket pump (TOUCH; SKC Ltd, Blandford Forum, UK) provided a flow of 125 ml min^−1^ through the LI‐840A, where data were recorded every 5 s.

Raw PTR–ToF–MS data were processed using the PTRwid software tool (Holzinger, 2015). PTRwid detected peaks in the measured spectra, applied a mass scale calibration, and subsequently, calculated the mixing ratios (nmol mol^−1^) of the identified BVOCs.

We obtained a mass range of *m*/*z* 18–237, and masses below *m*/*z* 33 were excluded from the dataset. Six focus compounds were selected based on their high flux rates and known importance in ecosystem‐atmosphere exchange, and attributed to these *m*/*z* ratios: methanol (*m*/*z* 33.029), acetic acid (sum of *m*/*z* 43.013 and *m*/*z* 61.031), acetaldehyde (*m*/*z* 45.026), and acetone (*m*/*z* 59.051), all of which are short‐chained oxygenated BVOCs, and isoprene (*m*/*z* 69.069) and monoterpenes (sum of *m*/*z* 81.065 and *m*/*z* 137.122), which are terpenoids (Yáñez‐Serrano et al., 2021).

The PTR–ToF–MS was calibrated three times during the experiment: before the start, after the pre‐flooding experiment, and at the end of the whole experiment with a gas standard (Ionicon Analytic, Innsbruck, Austria) containing a mixture of several VOCs at ppmv‐levels, diluted in nitrogen using a liquid calibration unit (Ionicon, Innsbruck, Austria). Five of the six focus compounds (methanol, acetaldehyde, acetone, isoprene, and α‐pinene) in the gas standard were used to directly calibrate the fluxes of the PTRwid output. α‐Pinene was used as a proxy for total monoterpenes. The gas calibration standard did not include acetic acid, and this compound was quantified using the PTRwid output based on an instrument transmission function and its known protonation reaction rate constant (Cappellin et al., 2012).

### 2.6 BVOC and CO_2_ Data Processing

A dead‐time of 15 s after valve‐switching from one mesocosm to another was excluded from the analysis to ensure the line from the valve to the infrared gas analyzer and PTR–ToF–MS (ca. 200 cm, 1/8 PFA) had been flushed. To obtain a net change in mixing ratios of BVOCs and CO_2_, an interpolated average mixing ratio in the blank chambers, measured before and after each mesocosm measurement, was subtracted from the mixing ratios at the outlet of the chambers. This net change was multiplied with the inflow rate, then divided by the mesocosm area (0.032 m^2^), to get a net flux per square meter of soil, resulting in one calculated flux from each mesocosm per hour. A positive flux value indicates net release and negative flux indicates net uptake to the ecosystem for both the CO_2_ and BVOC data.

During each experiment (pre‐flooding and flooding), early and late periods containing hourly averages from the five mesocosm replicates across 2–4 days were defined (Figure 2). The two warmest hours (hours 15 and 16) and the two coldest hours (hours 3 and 4) were selected as representative of “Day” and “Night”, respectively (Figure S3 in Supporting Information S1). Subsequently, the average of these Night and Day hours (*n* = 5) were used for statistical analyses.

### 2.7 Statistical Analysis

The R language (R Core Team, 2020) was used for statistical analyses. An analysis of variance (ANOVA) linear mixed effect model (lme4), was used to analyze for the effects of climate scenarios (ambient, cooling_dark, and warming), flooding (pre‐flooding and flooding), and period (early and late) on the fluxes of methanol, acetic acid, acetaldehyde, acetone, isoprene, and monoterpenes, as well as CO_2_. The Night (averages of the hours 3–4) and Day (averages of the hours 15–16) measurements were used separately to assess the effects of the climate scenarios, flooding, and period. Climate scenario, flooding, and period, together with all possible interactions, were fixed factors, and the corresponding background mixing ratio for the specific BVOC (nmol mol^−1^) compound or CO_2_ (ppmv) was a covariate to account for potentially high concentration gradients. A random factor was added to account for the repeated measurements on the mesocosms pre‐flooding and flooding. The mesocosm IDs were also included as a random factor to account for unobserved heterogeneity. The model was reduced stepwise starting with the highest interaction and highest *P*‐value using a threshold of *P* > 0.2. All statistical significances are presented in Table S5 and S6 in Supporting Information S1. There were no effects of having the lights periodically on (March 20 and 25, and April 3 and 8) in the cooling dark climate scenario and as such, this data will not be discussed further and all statistical comparisons herein include only cooling_dark periods where the lights were off.

A linear model was performed on the soil parameters, greenness, and gravimetric soil water content to test for significant differences among the climate scenarios (ambient, cooling_dark, and warming), flooding (pre‐flooding and flooding), and time (measurement days of the particular variable). If a significant difference was found among the climate scenarios, a Dunnett's post hoc test was applied with the ambient climate scenario as the control. Tukey's post hoc test was used to test for significant differences between interactions of pre‐flooding/flooding and early/late periods.

We obtained parameter estimates between the climate scenarios, flooding treatments, and periods from estimated marginal means (EMMs) using the default/balanced “emmeans” function from the *emmeans* package (Lenth, 2020). We present our results as EMMs rather than unadjusted means based on raw data, because EMMs correspond to the statistical models and account for covariates and potential imbalances in the dataset.

To assess how the climate scenarios and flooding affected the compound composition, a principal component analysis (PCA) was performed using SIMCA 16.0.1 (Umetrics, Umeå, Sweden). The PCA was conducted on unit variance scaled BVOC flux data averaged for day and night phase, early and late period, and pre‐flooding and flooding.

## 3 Results

### 3.1 Plant Species Composition and Greenness

Evergreen and deciduous shrubs dominated the plant species composition, with litter and moss covering the ground layer (Table S3 in Supporting Information S1). *Betula nana* L., *Vaccinium uliginosum* L., *Andromeda polifolia* L., and *Empetrum hermaphroditum* (Hagerup) Böcher dominated the plant species across all climate scenarios and there were no substantial differences in plant species composition (Table S3 in Supporting Information S1).

Generally, the greenness decreased throughout the experiment in the ambient and cooling_dark climate scenarios, whereas there was an increase in the warming (*P* < 0.005, Table S7 in Supporting Information S1), a trend that was applicable for both pre‐flooding and flooding periods. Flooding had no significant effect on the greenness.

### 3.2 Soil Parameters

Of the soil characteristics we examined, only the PO_4_^3−^ concentrations were affected by the climate scenarios, with significantly higher concentrations in the warming compared to the ambient and cooling_dark scenarios (Table 1, *P* < 0.05).

**Table 1:** pH and Concentrations of Extractable Carbon and Nitrogen in the Mesocosm Soil (Mean ± SE, n = 5)

	Start	Pre‐flooding			Flooding		
Climate scenario		Ambient	Cooling_dark	Warming	Ambient	Cooling_dark	Warming
DOC (μg g^−1^ d.w.)	729 ± 126	673 ± 81	713 ± 106	755 ± 87	853 ± 77	641 ± 95	722 ± 94
TN (μg g^−1^ d.w.)	37.7 ± 5.3	36.2 ± 3.9	50.8 ± 6.7	45.5 ± 5.6	48.2 ± 5.1	41.3 ± 7.1	45.9 ± 6.4
NO_3_^−^ (μg g^−1^ d.w.)	2.9 ± 0.9	2.7 ± 0.2	4.6 ± 1.1	4.7 ± 1.4	1.3 ± 0.2	1.0 ± 0.1	2.8 ± 0.9
NH_4_^+^ (μg g^−1^ d.w.)	11.0 ± 3.2	5.4 ± 1.3	13.1 ± 3.2	9.7 ± 2.7	12.1 ± 3.2	17.3 ± 4.7	12.1 ± 2.7
PO_4_^3−^ (μg g^−1^ d.w.)	4.9 ± 2.5	4.1 ± 1.5	2.0 ± 0.9	12.0 ± 4.2	5.7 ± 1.7	2.1 ± 0.6	10.7 ± 2.1
pH	6.1 ± 0.3	6.2 ± 0.1	6.4 ± 0.2	6.2 ± 0.2	6.6 ± 0.2	6.7 ± 0.2	6.4 ± 0.1

*Note.* The concentrations were measured before initiation of the experiment (Start), then after the pre‐flooding experiment (Pre‐flooding), and at the end of the flooding experiment (Flooding) for each climate scenario (Ambient, Cooling_dark, and Warming). DOC = dissolved organic carbon; TN = total dissolved nitrogen; NO_3_^−^ = nitrate; NH_4_^+^ = ammonium; PO_4_^3−^ = phosphate.

Flooding significantly decreased the NO_3_^−^ concentrations (*P* = 0.011) and pH tended to increase from 6.3 pre‐flooding to 6.6 when flooded (*P* < 0.1, across all climate scenarios, Table 1).

### 3.3 Net Ecosystem Exchange of CO_2_

**Figure 3:**
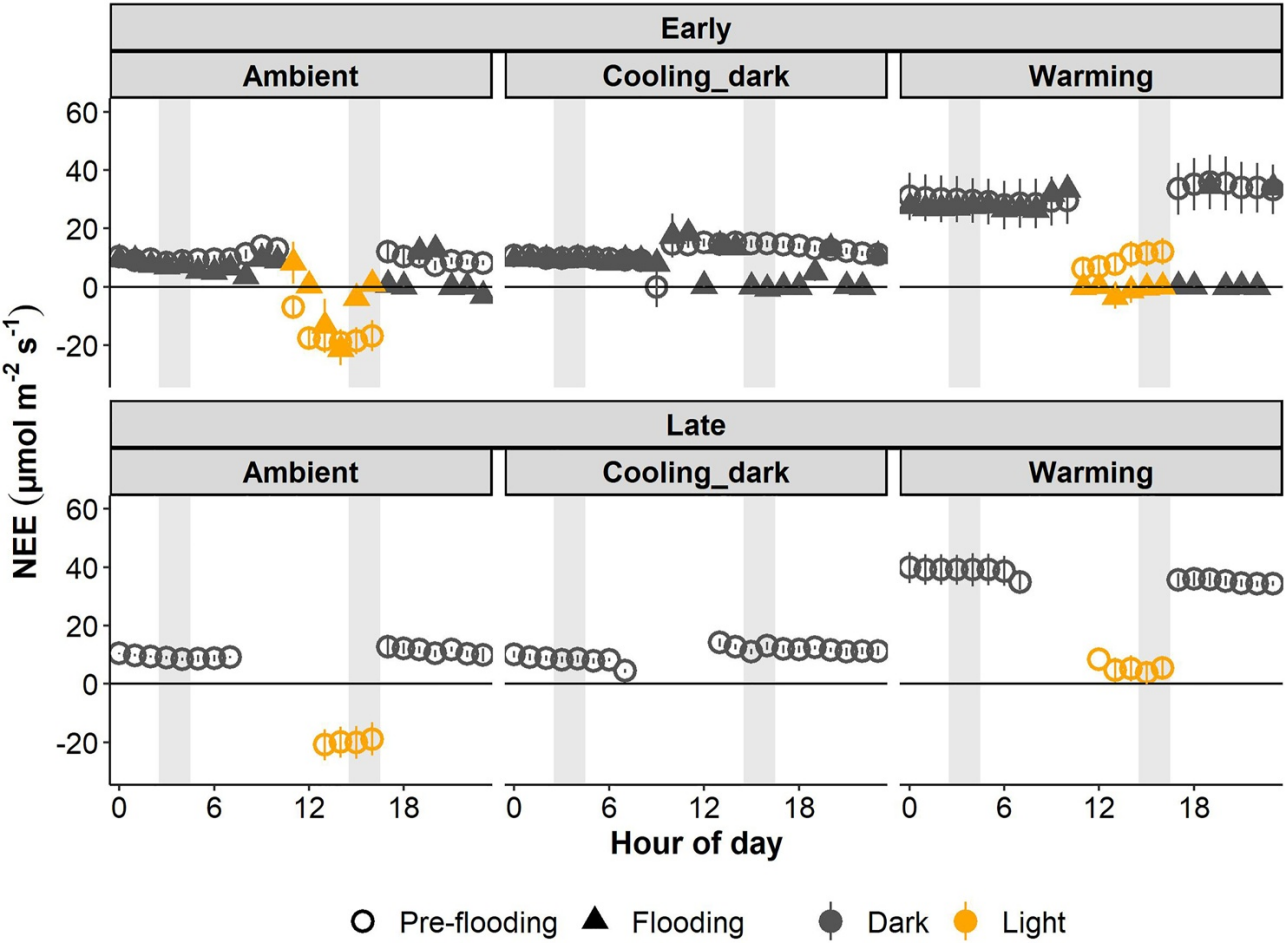
Net ecosystem exchange (NEE) of CO_2_ for each climate scenario pre‐flooding (circles) and flooding (triangles). Early and late show periods at the beginning and end of each experiment, respectively. Symbols show the mean ± SE, *n* = 5. Positive values depict net release from and negative values net uptake into the mesocosms. Shaded gray bars illustrate data used in the statistical analyses. Data for the late period with flooding was not recorded due to unavailability of the instrumentation.

Throughout the experiment, NEE of CO_2_ followed the light conditions, with ecosystem respiration dominating when the lights were off and net CO_2_ uptake dominating when the lights were on for both the ambient and cooling_dark climate scenario (Figure 3 and Figure S4 in Supporting Information S1). The warming climate scenario mainly showed net CO_2_ release, which decreased during light periods.

The warming treatment showed a net CO_2_ release during the night, which was more than a 100% higher than in the ambient and cooling_dark scenarios (Figure 3). The light periods resulted in lower net respiration (warming) or uptake of CO_2_ (ambient). There were no differences between night and day in the cooling_dark treatment. Flooding caused an NEE of CO_2_ around zero between the hours of 18–24, regardless of the magnitude of release pre‐flooding, whereas hours 0–12 were essentially the same as for pre‐flooding (Figure 3).

**Figure 4:**
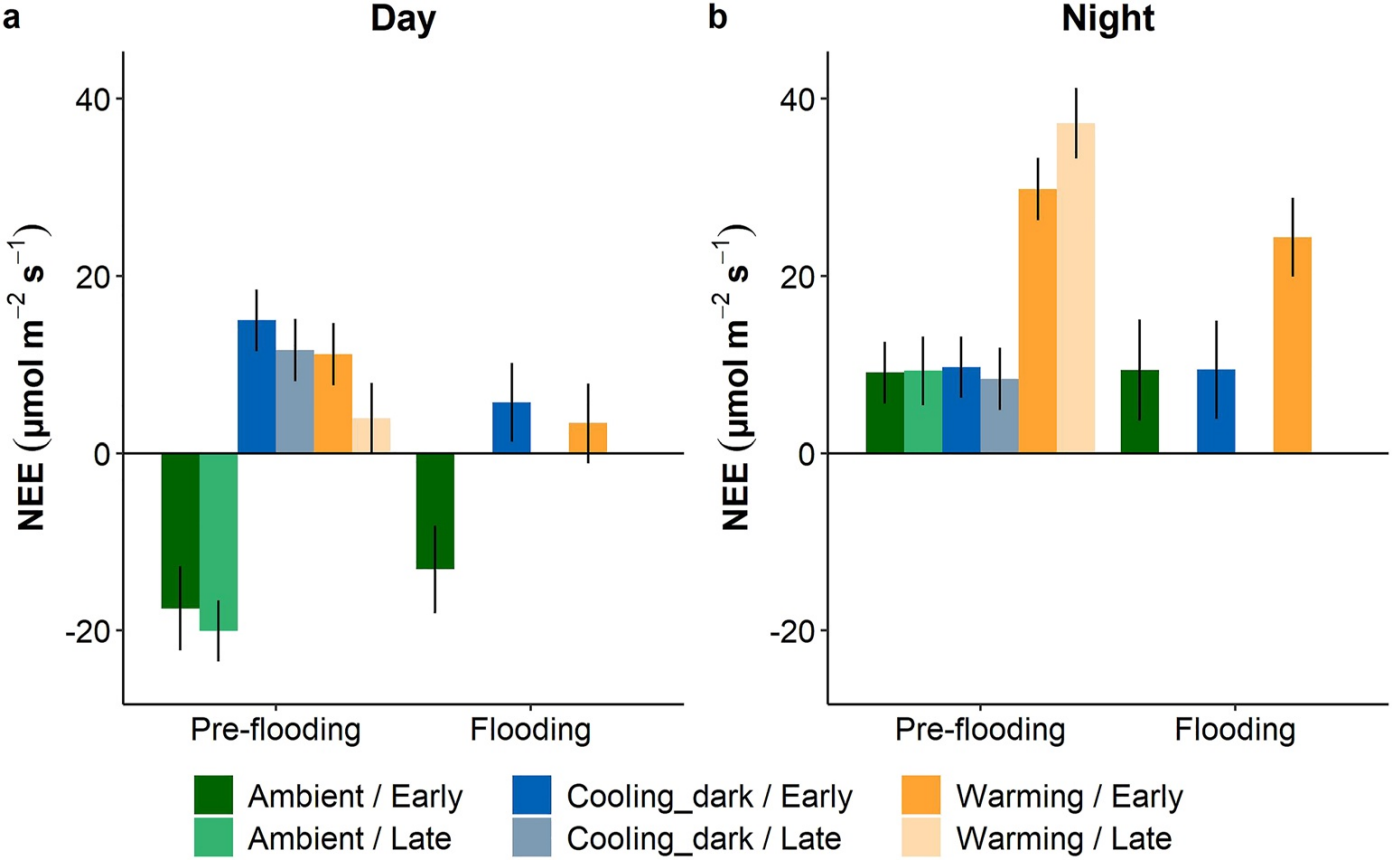
Estimated marginal means (EMM) for net ecosystem exchange (NEE) of CO_2_ during the warmest (15–16 = Day) and coldest (3–4 = Night) hours for each climate scenario, pre‐flooding and flooding, respectively. Early and late show periods in the beginning and end of the experiment, respectively. Bars show mean ± SE, *n* = 5. Positive values depict net release from and negative values indicate net uptake into the mesocosms. No NEE of CO_2_ data are available for late period in the Flooding experiment.

Overall, the EMMs for net CO_2_ exchange were higher during night than during the day. When looking at only the daytime NEE of CO_2_, the ambient scenario displayed net uptake of CO_2_ and differed significantly from the cooling_dark and warming scenarios, which showed net release (*P* < 0.005, Figure 4) in both the pre‐flooding and flooding experiments. In contrast, the warming scenario exhibited significantly higher emissions of CO_2_ compared to the ambient and cooling_dark scenarios during the night (*P* < 0.001). Flooding reduced the CO_2_ release significantly during day in the warming‐ and cooling_dark climate scenarios (*P* < 0.05), whereas the night was unaffected by flooding.

### 3.4 BVOC Composition

The mesocosm BVOC fluxes were dominated by net uptake of acetic acid, methanol, and acetone. The 10 compounds with the highest net uptake rates accounted for ≈75% (molar) of the total fluxes measured, whereas the 10 compounds with the highest net release only accounted for ≈5% (molar) of the absolute total of uptake and release. Compounds with mass ranges below 80 a.m.u. dominated the uptake, whereas compounds in the mass range of 80–150 a.m.u. dominated the release (Table 2). Methanol, acetic acid, acetone, and acetaldehyde were all represented in the top 10 net negative fluxes.

**Table 2:** Fluxes for the 10 Protonated Masses (and Tentative Compound Identification, Where Applicable) With the Highest Release and Uptake Rates (Mean ± SE Across All Climate Scenarios and Periods, n = 15)

	Protonated mass (*m*/*z*)	Flux (nmol m^−2^ s^−1^)	Range (min–max)
**Top 10 net release compounds**	112.032	0.110 ± 0.008	−0.2 to 11.2
	84.076	0.022 ± 0.002	−0.1 to 1.5
	39.953	0.013 ± 0.001	−0.2 to 0.2
	123.038	0.013 ± 0.001	−1.3 to 1.2
	63.017	0.011 ± 0.001	−0.5 to 0.7
	100.024	0.009 ± 0.001	−0.1 to 2.5
	133.048	0.0070 ± 0.0004	−0.2 to 0.2
	153.054	0.0050 ± 0.0002	0 to 0.1
	111.951	0.0050 ± 0.0004	−0.1 to 0.5
	40.962	0.0040 ± 0.0004	−0.1 to 0.2
	84.049	0.0040 ± 0.0004	−0.01 to 0.4
**Top 10 net uptake compounds**	*33.029 (methanol)*	−1.260 ± 0.024	−7.6 to 8.6
	*43.013 and 61.028 (acetic acid)*	−1.040 ± 0.034	−29 to 44
	47.012	−0.730 ± 0.013	−7.8 to 8.6
	93.067	−0.540 ± 0.007	−2.3 to 2.4
	*59.051 (acetone)*	−0.520 ± 0.011	−5.2 to 3.3
	42.029	−0.410 ± 0.006	−3.5 to 1.3
	73.061	−0.410 ± 0.004	−2.1 to 2.1
	75.044	−0.370 ± 0.015	−9.9 to 17
	*45.026 (acetaldehyde)*	−0.270 ± 0.005	−2 to 1.8
	89.039	−0.180 ± 0.007	−3.7 to 7.5

*Note.* Range = minimum and maximum flux measured. Focus compounds are in *italics*.

#### 3.4.1 Timeline and Averaged 24 hr Fluxes

**Figure 5:**
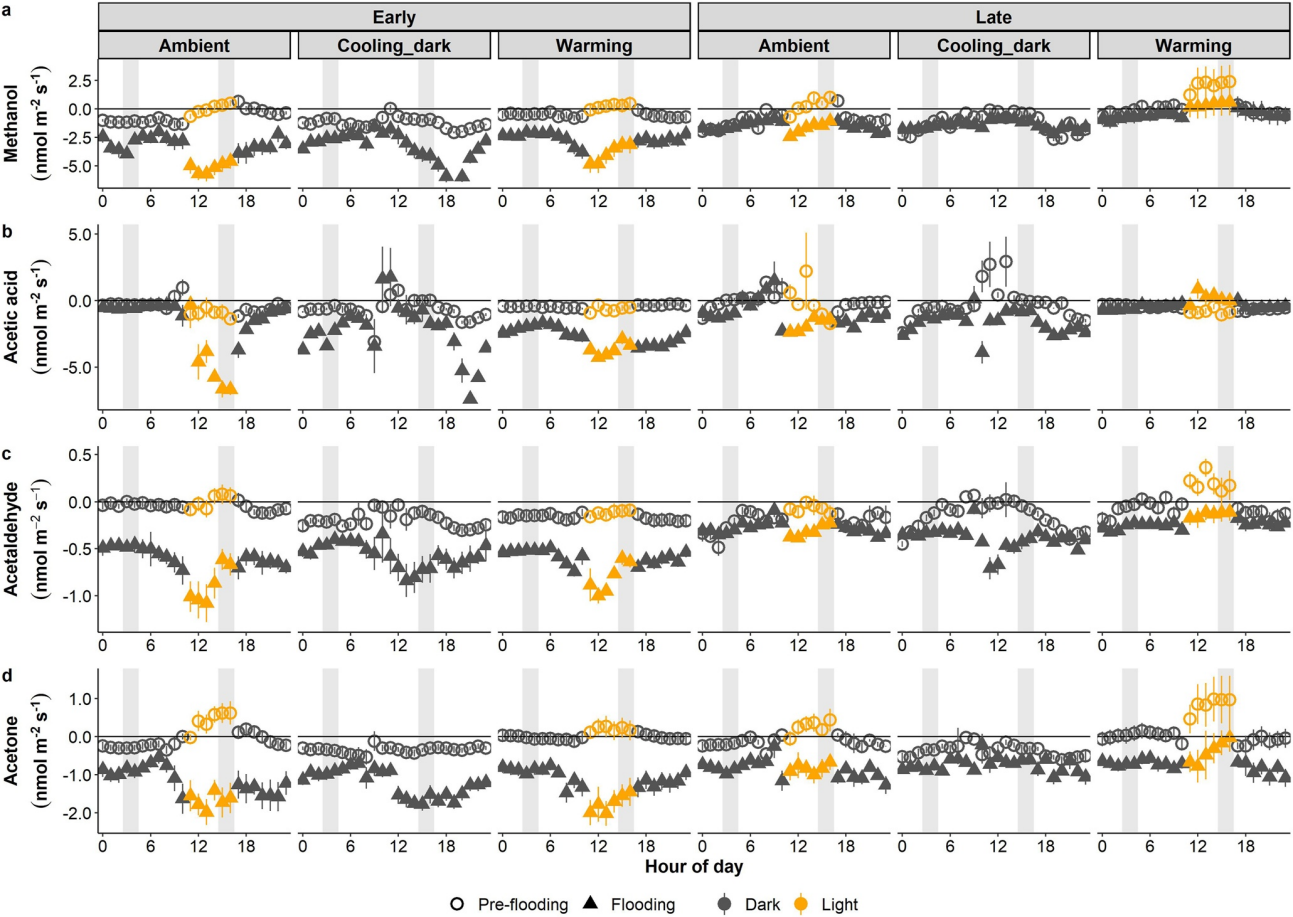
Methanol (a), acetic acid (b), acetaldehyde (c), and acetone (d) diurnal fluxes for each climate scenario pre‐flooding and flooding, respectively. Early and late show periods at the beginning and end of each experiment, respectively. Symbols show the mean ± SE, *n* = 5. Positive values depict net release from and negative values indicate net uptake into the mesocosms. Shaded gray bars illustrate data used in the statistical analyses.

**Figure 6:**
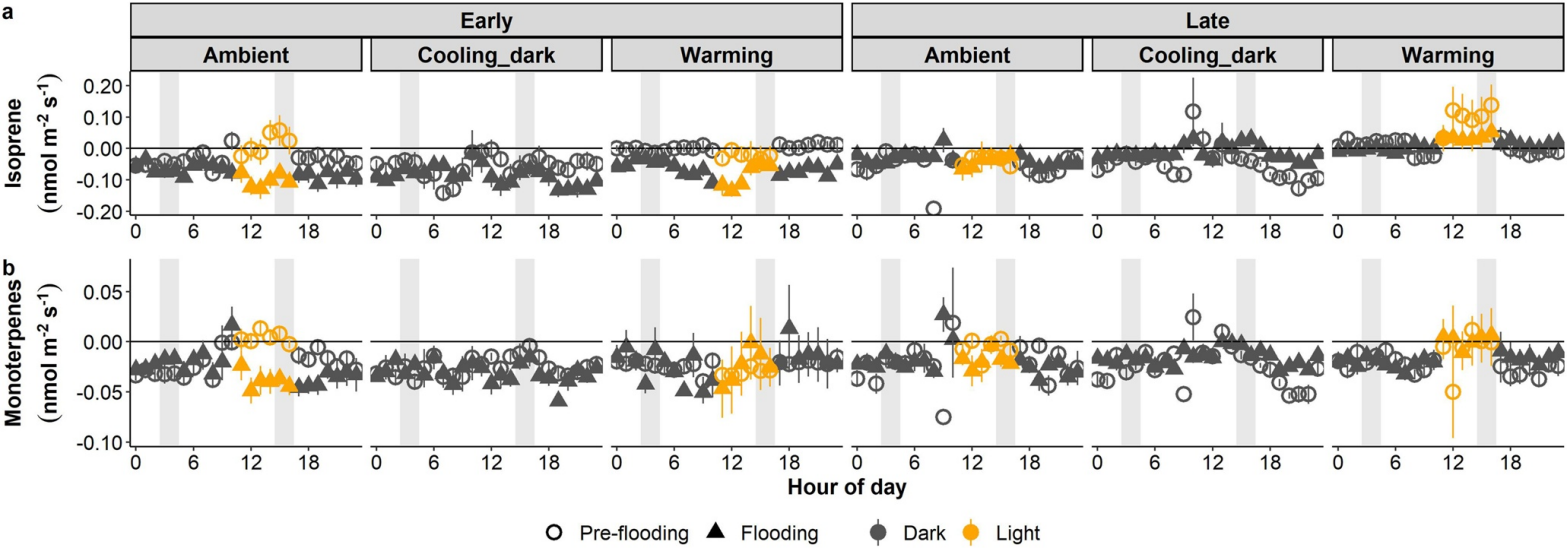
Isoprene (a) and monoterpene (b) diurnal fluxes for each climate scenario pre‐flooding and flooding, respectively. Early and late show periods at the beginning and end of each experiment, respectively. Symbols show the mean ± SE, *n* = 5. Positive values depict release from and negative values indicate uptake into the mesocosms. Shaded gray bars illustrate data used in the statistical analyses.

Over the entire experimental period, fluxes of the four short‐chained oxygenated BVOCs (methanol, acetic acid, acetaldehyde, and acetone) generally fluctuated around zero pre‐flooding (Figure 5, Figures S6, S8, S10, and S12 in Supporting Information S1). When flooded, fluxes for the same four compounds were dominated by net uptake, which tended to decrease gradually toward zero across the flooding period (Figure 5, Figures S6, S8, S10, and S12 in Supporting Information S1). The terpenoid fluxes (isoprene and monoterpenes) fluctuated around zero during both the pre‐flooding and flooding experiments (Figure 6, Figures S14 and S16 in Supporting Information S1).

Pre‐flooding, the short‐chained oxygenated BVOC fluxes followed similar diel patterns across climate scenarios, with net methanol, acetaldehyde, and acetone uptake during the night compared to net release during the day in the ambient and warming scenarios (Figures 5a, 5c and 5d). In the cooling_dark scenario with no light, net uptake of all six focus compounds dominated throughout the day and night. For acetic acid, the mesocosms were a constant sink, except for two times in the late period ambient and cooling_dark climate scenarios, respectively (Figure 5b).

Flooding increased the net uptake for all four short‐chained oxygenated BVOCs across all climate scenarios and periods. Furthermore, net rates of daytime uptake increased in the ambient and warming scenarios when flooded, which was in contrast to the net releases observed during daytime pre‐flooding (Figure 5).

Isoprene showed net release during daytime in the ambient (early period) and warming (late period) pre‐flooding, but otherwise remained as net uptakes (Figure 6a). When flooded, isoprene fluxes switched to net uptake during the daytime, but there was no change in fluxes during the night, relative to pre‐flooding. Monoterpenes generally exhibited net uptake with no clear effect of flooding (Figure 6b).

### 3.5 Fluxes During the Warmest (Day) and Coldest (Night) Phases

**Figure 7:**
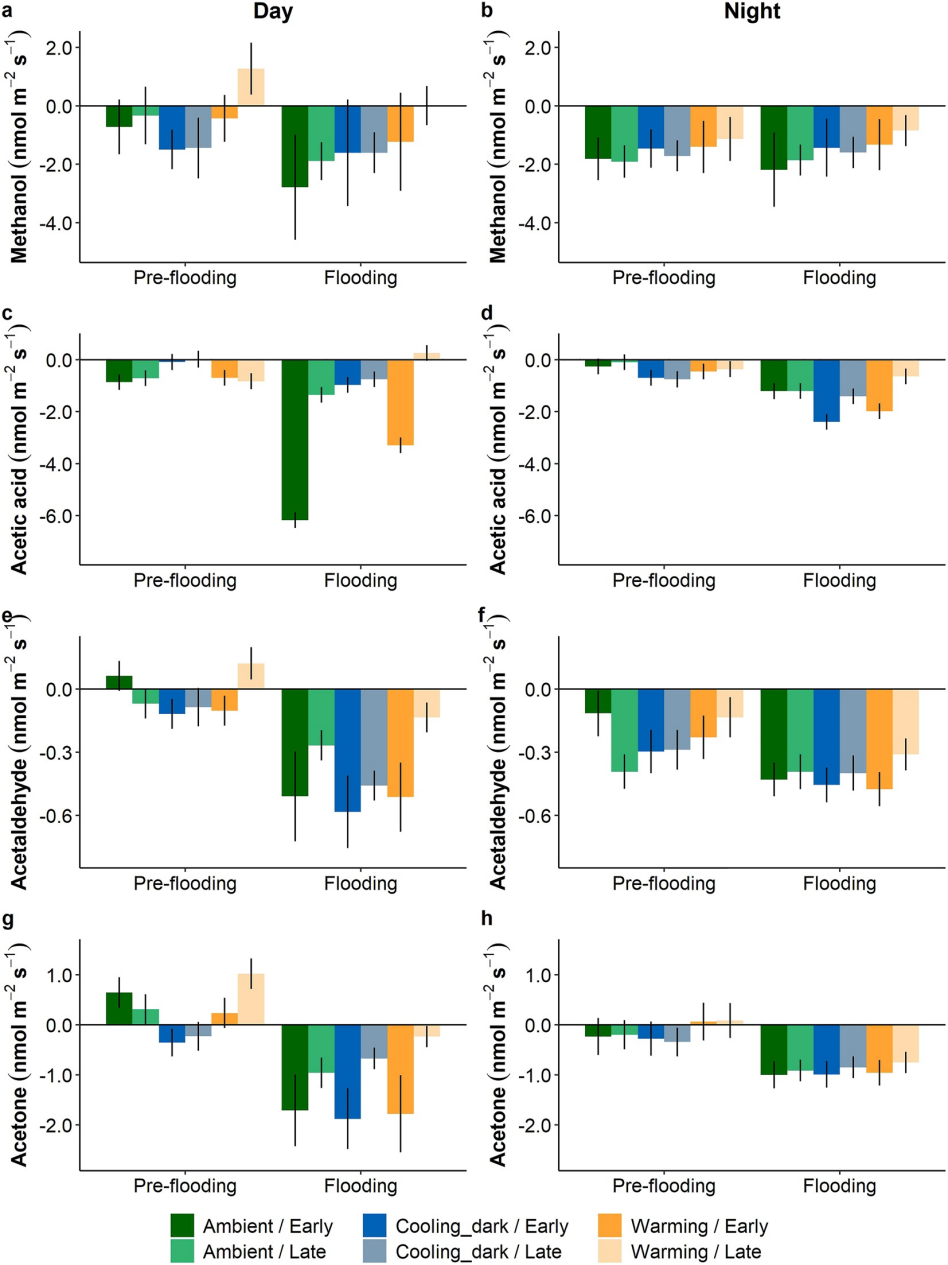
Estimated marginal means for methanol (a and b), acetic acid (c and d), acetaldehyde (e and f), and acetone (g and h) fluxes during the warmest (15–16 = Day) and coldest (3–4 = Night) hours for each climate scenario, pre‐flooding and flooding, respectively. Early and late show periods at the beginning and end of each experiment, respectively. Positive values depict release from and negative values indicate uptake by the mesocosms. Bars show mean ± SE, *n* = 5. Note the different *y*‐axis scales.

**Figure 8:**
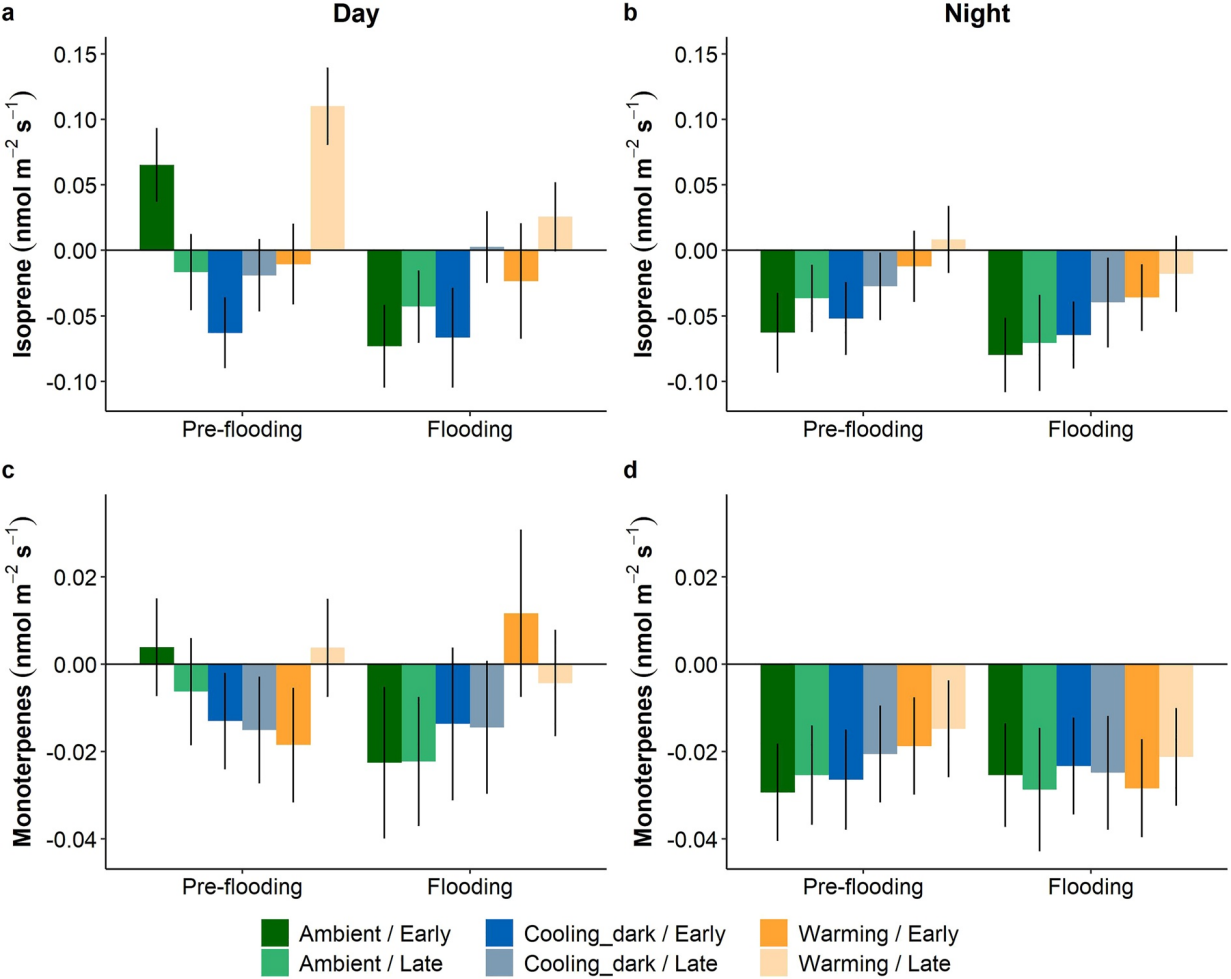
Estimated marginal means for isoprene (a and b), and monoterpene (c and d) fluxes during the warmest (15–16 = Day) and coldest (3–4 = Night) hours for each climate scenario, pre‐flooding and flooding, respectively. Early and late show periods at the beginning and end of each experiment, respectively. Positive values depict release from and negative values indicate uptake into the mesocosms. Bars show mean ± SE, *n* = 5. Note the different *y*‐axis scales.

Overall, there were clear effects of the different factors tested (climate scenarios, flooding, and period) on the six focus compounds when looking at the day phase (Figures 7 and 8). For all six compounds, the net flux during the night occurred predominantly as uptake, whereas day measurements varied between net positive and negative exchanges, depending on the different factors and compounds (Figures 7 and 8).

Warming significantly affected the methanol fluxes (*P* < 0.05). During the day, the net flux across pre‐flooding and flooding was close to zero, whereas the ambient and cooling_dark scenarios showed considerable net uptake (Figure 7a). During the night, net methanol uptake under warming was significantly lower than in the ambient climate scenario (Figure 7b). Methanol was unaffected by flooding and showed no significant differences between the early and the late period.

For acetic acid, there was a strong interaction effect between climate scenarios, flooding, and period during both the day and night. The climate scenarios only affected acetic acid fluxes significantly after the flooding. During the day, the warming and cooling_dark climate scenarios resulted in lower net uptake in the early period compared to the strong net uptake observed in the ambient climate scenario (*P* < 0.001, Figure 7c). In the late period, warming had a net release of methanol, whereas the ambient scenario exhibited a net uptake (*P* < 0.05). During night in the early period, the cooling_dark scenario had a significantly higher uptake than the ambient scenario (*P* < 0.05, Figure 7d). Flooding also had a significant effect on acetic acid fluxes. Both warming and ambient net uptake in the early period were significantly higher when flooded compared to the pre‐flooding fluxes (*P* < 0.001). The net flux in the late period in the warming scenario on the other hand, shifted from a net uptake pre‐flooding to a net release when flooded (*P* < 0.05). During the night, all acetic acid fluxes exhibited increased net uptake after flooding (*P* < 0.05), except during the late period of the warming scenario, which did not differ significantly. Warming and ambient climate scenarios differed significantly between the daytime early and late periods when flooded, whereby the acetic acid flux under warming switched from a net uptake in the early period to a net release in the late period, while the net uptake in the ambient scenario decreased significantly in the late period (*P* < 0.001). During the night, cooling_dark and warming net uptake decreased significantly in the late period compared to the early period (*P* < 0.05).

Acetaldehyde was affected by the climate scenarios during the night, pre‐flooding (Figure 7f). In the early period of the cooling_dark scenario, the uptake was significantly higher than in the ambient scenario (*P* < 0.05), whereas warming in the late period had a significantly lower uptake than in the ambient scenario (*P* < 0.001). During the night in the early period, flooding significantly increased the net uptake in the ambient and warming scenarios (*P* < 0.05), whereas flooding in the late period significantly increased net uptake in the ambient and cooling_dark scenarios (*P* < 0.05). There were no significant differences between the early and late periods during the day. However, during the night pre‐flooding, the ambient net uptake increased significantly in the late period relative to the early period (*P* < 0.001), whereas the warming net uptake decreased in the late period in both pre‐flooding and flooding experiments (*P* < 0.05).

Acetone was affected by the climate scenarios during the day (Figure 7g). In the pre‐flooding experiment, the cooling_dark scenario showed a net uptake, whereas the ambient scenario exhibited a net release (*P* < 0.05). However, in the late period, the cooling_dark scenario had a net release, while the ambient scenario showed a net uptake (*P* < 0.05). Flooding switched all positive fluxes to negative fluxes or increased the net uptake significantly, compared to the pre‐flooding experiment, both during the day and night (*P* < 0.05, Figures 7g and 7h). There was only a significant difference between periods during the day. Under the warming scenario, the net uptake of acetone in the early period switched to a net release in the late period (*P* < 0.05), and the net uptake observed in the cooling_dark scenario early period decreased significantly in the late period (*P* < 0.05).

Isoprene was affected by the climate scenarios during the day in the late period where warming caused a net release of isoprene, as opposed to the net flux close to zero in the ambient scenario (Figure 8a, *P* < 0.05). At night, the warming scenario exhibited a significantly lower isoprene net uptake than in the ambient scenario (Figure 8b, *P* < 0.05). Flooding resulted in a net isoprene uptake in the ambient scenario, whereas the pre‐flooding conditions had a net release during the day (*P* < 0.05). At night, flooding increased the overall net uptake (*P* < 0.05). Monoterpenes were not significantly affected by any of the factors tested (Figures 8c and 8d).

### 3.6 Climate Scenario Effects on the BVOC Composition

**Figure 9:**
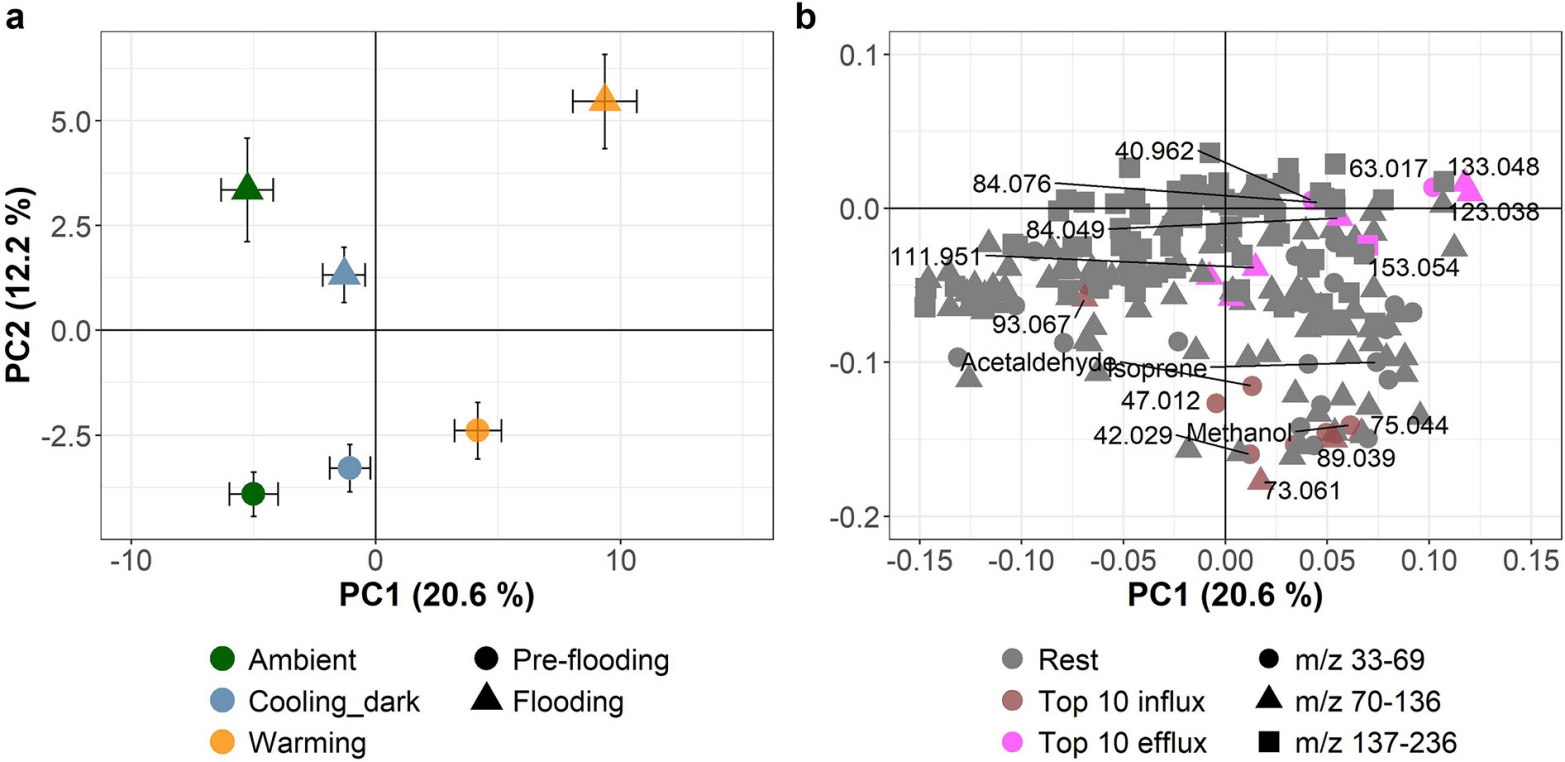
Principal component analysis of the flux data for 216 individual masses. (a) Scores (mean ± SE, *n* = 5) for each climate scenario (ambient, cooling_dark, and warming), pre‐flooding, and flooding for principal components (PC) 1 and 2. (b) Corresponding loadings for the masses divided into three groups based on their *m*/*z* ranges. Variances explained by the PCs are shown in parentheses.

The PCA revealed that the BVOC composition was altered by both the climate scenarios and flooding (Figure 9a). The first principal component (PC), which explained 20.6% of the variance, described the differences between the climate scenarios, with the warming scenario having the highest scores along this axis and the ambient scenario exhibiting the lowest scores. The second PC, which explained 12.2% of the variance, divided the samples between flooding and pre‐flooding.

The 20 compounds with the highest flux rates were generally more characteristic of the warming scenario than the ambient scenario (Figure 9b). Pre‐flooding, the characteristic compounds for the warming climate scenario included the masses with the highest uptake rates, while when flooded, warming had higher contributions from *m*/*z* 63.017, 123.038, and 133.048, which were among the compounds with the highest release rates. Flooding caused the ambient climate scenario to shift from a compound composition including compounds from all mass ranges to a composition dominated by *m*/*z* 137–236.

## 4 Discussion

In this work, we simulated three subarctic autumn climate scenarios to assess how the bidirectional BVOC fluxes would behave under possible future conditions. We selected six focus BVOCs (methanol, acetic acid, acetaldehyde, acetone, isoprene, and monoterpenes) based on their high flux rates and known importance in ecosystem‐atmosphere exchange. Generally, the fluxes of all the focus BVOCs and CO_2_ were affected by the different climate scenarios. Warming enhanced the vegetation growth and soil respiration, and resulted, more often, in net release than was the case for the cooling_dark scenario. The cooling_dark climate scenario was mainly dominated by net BVOC uptake and low vegetation activity. These generalizations applied to most of the focus BVOCs. Monoterpenes were the only compound group that was unaffected by all of the tested factors. Flooding resulted in increased net uptake for most of the focus compounds and a simultaneous decrease in net CO_2_ release.

### 4.1 Warming Increased BVOC Net Release

Warming increases the activity of all biological processes, below an optimal temperature threshold (Baggesen et al., 2021; Mörsdorf et al., 2019; Semenchuk et al., 2016). We also found a significantly higher net CO_2_ release under warming compared with the ambient climate scenario (Figure 4), indicating that ecosystem respiration (inferring the same respiration rates occurred during the day as at night) increased (Baggesen et al., 2021; Don & Kalbitz, 2005). Soil respiration rates are closely linked to soil BVOC production rates (Leff & Fierer, 2008). As hypothesized, the fluxes of methanol, acetic acid, acetaldehyde, acetone, and isoprene were all significantly affected by warming (Figures 7 and 8), resulting in more positive net fluxes for all five compounds, either as a net release or a reduced net uptake. Besides the direct effect of warming on the fluxes, indirect vegetation‐related changes may also have altered the fluxes (Valolahti et al., 2015); we found a significant increase in greenness during the experimental period in the warming climate scenario, suggesting increased photosynthetic activity, while greenness in the other climate scenarios largely declined. For example, methanol is released from leaves as a by‐product of pectin methylesterase activity during leaf growth and expansion (Fall 2003), and is thus, an indicator of growth, which supports our results of increasing methanol release when temperature and greenness increased. Furthermore, we observed a significantly lower methanol net uptake (close to zero) during the day, compared to at night, which further supports the suggestion that some of the methanol production may originate from leaf growth and the release via stomata during daylight periods (Hüve et al., 2007). Furthermore, methanol, as well as acetaldehyde, is released from a wide variety of decomposing leaf litter types (Gray et al., 2010), which might be enhanced by the higher temperatures in the warming climate scenario. Besides being produced during litter decomposition, the short‐chained oxygenated BVOCs (e.g., methanol, acetic acid, acetaldehyde, and acetone) can also serve as a carbon source for microbes (Albers et al., 2018). Thus, the net flux depends on the balance between release and uptake.

Isoprene is formed by the methylerythritol 4‐phosphate pathway, a part of photosynthesis, and is released immediately (Loreto & Schnitzler, 2010). Thus, isoprene emissions are highly dependent on PAR and temperature. Furthermore, warming‐induced increases in soil PO_4_^3−^ concentrations could support higher plant growth and together with increased vegetation greenness, are in line with the significant increase in isoprene net release observed in the late period in the warming climate scenario, relative to the ambient scenario. During the night when plant isoprene production was inactive, net uptake of isoprene was significantly lower under warming as compared to the ambient climate scenario (Figure 8). This suggests differences in microbial isoprene production or uptake (McGenity et al., 2018) that result in altered net flux. Rinnan et al. (2013) showed that most BVOCs emitted during autumn in a subarctic, mixed heath were produced by plants, but increased temperatures under warming might change the production‐balance towards higher microbial contributions.

### 4.2 Cooler and Darker Autumn Conditions Are Dominated by Net BVOC Uptake

The cooling_dark climate scenario was characterized by net uptake for all six focus compounds. The low temperatures and lack of light, in combination with increasingly frozen soil, resulted in low ecosystem activity, in terms of both plant growth and microbial decomposition. Low temperatures constrain the diffusivity of the cell membrane resulting in decreased transport across membranes (Sung et al., 2003). The lack of light in the cooling_dark scenario inhibited photosynthesis and consequently, the vegetation activity was low. As such, the CO_2_ and BVOC fluxes were mainly a result of microbial activity. BVOCs may be produced by microbes under very low temperatures (Aaltonen et al., 2012), but this production might be hidden by BVOC sinks, such as abiotic deposition due to the cold temperatures (Niinemets et al., 2014), microbial BVOC uptake (Albers et al., 2018), and produced BVOCs which are not released, due to entrapment in ice layers (Kramshøj et al., 2018). Furthermore, the vegetation will only add minor contributions to the microbial BVOC production (e.g., via root exudates) due to the lack of light to allow for plant productivity.

We expected the light‐dependent BVOCs (methanol, acetone, isoprene, and monoterpenes) to be affected by the cooling_dark climate scenario (Fall, 2003; Huang et al., 2018; Loreto & Schnitzler, 2010), due to the lack of light during the day phase (in contrast to the ambient and warming scenarios). Contrary to our expectations, we only saw significant effects on acetic acid, acetaldehyde, and acetone mainly as an increased net uptake or a switch from net release to net uptake, but the responses also interacted with flooding and period.

Acetic acid is rapidly incorporated into soil microbes, and thus, provides an indication of microbial growth in the ecosystem (Rinnan & Bååth, 2009). As such, the lower net uptake of acetic acid in the cooling_dark scenario during the day could suggest less microbial uptake compared to the ambient scenario.

### 4.3 Flooding Resulted in BVOC Deposition

As hypothesized, most of the fluxes of the short‐chained oxygenated BVOCs (acetic acid, acetaldehyde, and acetone) were altered by flooding. These BVOCs are water soluble and thus, their plant‐atmosphere exchange is stomatal dependent and their soil‐atmosphere exchange is soil moisture dependent (Seco et al., 2007).

The flooding experiment was characterized by high BVOC uptake. Normally, wetting events are known to create instant bursts of BVOC release, evidenced by the characteristic smell from newly wetted ecosystems (Rossabi et al., 2018). Here, we focused on the BVOC fluxes under a constant flooded state, as flooding was performed while not monitoring gas fluxes. Microbial uptake of BVOCs is normally aerobic (Tang et al., 2019), but in the flooded state, the soils are anaerobic. Therefore, the high net uptake probably does not originate from microbial activity. It is more likely that the high net uptake of BVOCs was due to abiotic deposition and dissolution in the waterlogged soils. Especially short‐chained oxygenated BVOCs may get trapped at wet surfaces and additionally, their high water solubility makes them easily accessible for microorganisms at the aerobic/anaerobic interface (Tang et al., 2019).

Although acetaldehyde is water‐soluble, it was expected that this compound would exhibit a net release when flooded, because acetaldehyde is produced in flooded roots (Fall, 2003), and especially in species not adapted to waterlogged soils, like shrubs. Even though shrubs accounted for >50% of the total live biomass in this experiment, net uptake of acetaldehyde was still measured.

The net CO_2_ flux might not only be directly affected by the anaerobic conditions, but also by some indirect effects of flooding. Under anaerobic circumstances, denitrification occurs, resulting in a decrease in the available nitrogen in nitrate form (Alaoui‐Sossé et al., 2005), which could limit plant growth or microbial activity. In this experiment, we found a significant decrease in nitrate following the flooding experiment, compared to the pre‐flooding concentrations. Furthermore, the soil pH tended to increase after flooding, which may also affect ecosystem processes (Yao et al., 2009), and may be a result of reduction reactions, such as denitrification, where consumption of H^+^ results in increased pH (Narteh & Sahrawat, 1999). In addition, we hypothesized that the switch from aerobic to anaerobic conditions might change the BVOC composition to reflect fermentative processes under flooding (Rinnan et al., 2014). The PCA showed clear differences in BVOC compositions in the pre‐flooding and flooding experiments, which supports our hypothesis. For example, the ambient climate scenario shifted from being associated with compounds across all mass ranges pre‐flooding, to being dominated by *m*/*z* 137–236 when flooded.

Flooding significantly increased the net uptake of isoprene in the ambient climate scenario. Unlike the short‐chained oxygenated compounds, non‐oxygenated terpenoids are not water soluble (Weidenhamer et al., 1993). Thus, these compounds were not dissolved in the added water, which could explain why we do not see any effect of flooding on the monoterpene fluxes. Niinemets (2010) showed that mild stress‐effects on plants depend on the presence of carbon reserves that plants need to produce BVOCs (Peñuelas & Llusia, 2003), whereas acute stress normally leads to large reductions in BVOC fluxes. In our flooding experiment, we found a decrease in net CO_2_ flux, which shows that the microbial respiration was reduced during flooding. It is likely that microbial isoprene production and consumption (McGenity et al., 2018) were also inhibited under waterlogged conditions. Thus, the inhibition of microbial processes, together with the lack of isoprene storage in plants (Feng et al., 2019), and the fact that the isoprene‐emitting vegetation was stressed under waterlogged conditions, may explain why we see a significant decrease in isoprene release after flooding. In contrast, no effect of flooding was observed for the monoterpene fluxes, compounds which are known to be stored (Vanhatalo et al., 2018).

### 4.4 Climate Scenario Effects Differed Between the Early and Late Periods

As autumn approaches, the ecosystem prepares for winter; the vegetation enters winter dormancy and the soil microbial activity decreases, which both result in reduced CO_2_ and BVOC fluxes during the autumn transition period (Finderup Nielsen et al., 2019; Seco et al., 2011). Despite the relatively short duration of our experiment, we found a significant difference between the early and late periods for the fluxes of acetic acid, acetaldehyde, acetone, and isoprene where some increased and some decreased, whereas methanol, monoterpenes, and CO_2_ were unaffected.

Net CO_2_ assimilation and vegetation greenness were expected to decrease over time, due to the natural transition from summer productivity to winter dormancy (Christiansen et al., 2012; Ravn et al., 2020). The vegetation greenness in the ambient and cooling_dark climate scenarios showed a decreasing trend throughout the experiment, although the change was not statistically significant. The warming scenario, on the other hand, significantly increased in greenness throughout the experimental period. However, there were no significant differences in the CO_2_ fluxes between the early and late periods in the warming climate scenario, possibly because increased respiration compensated for any increase in CO_2_ assimilation.

Acetic acid, acetone, and isoprene are used as carbon sources for microbes (Albers et al., 2018) and the decreased uptake suggests that there is, in fact, less microbial activity during the late period. The frozen soil in the ambient and cooling_dark climate scenario have likely reduced soil microbial activity (Clein & Schimel, 1995). Acetone and isoprene in the warming scenario switched from a clear net uptake to a net flux around zero, or a net release, between the early and late periods. The switch was particularly clear in the pre‐flooding experiment. The decrease in net uptake could suggest that besides microbial uptake, there might be some BVOC production by photosynthetic activity of the vegetation (Niinemets et al., 2014), which is supported by the significant increase in greenness we observed. Greenness, warming, and changes in phenology are closely linked to BVOC fluxes (Baggesen et al., 2021; Lindwall et al., 2016; Tiiva et al., 2008) and these factors might explain the decrease in net uptake from the early to the late period. In the early green‐up, the short‐chained oxygenated BVOCs, such as acetone, are emitted (Baggesen et al., 2021), whereas isoprene is directly correlated with increasing temperatures and greenness (Lindwall et al., 2016; Loreto & Schnitzler, 2010; Tiiva et al., 2008).

Differences between the early and late periods for acetaldehyde varied between the climate scenarios. In the warming scenario during the day, the net uptake tended to decrease in the late period, showing the same pattern of net release as acetic acid, acetone, and isoprene. In the ambient (pre‐flooding, night) scenario, acetaldehyde uptake increased significantly between the early and late periods.

## 5 Conclusions

Autumn fluxes of the six focus BVOCs and CO_2_ were affected by changing temperatures and soil flooding. A warmer autumn may result in increased net emissions of BVOCs and CO_2_, as well as increased vegetation greenness. A cold and dark autumn will constrain the ecosystem productivity because of reduced light availability and frozen soil. Thus, BVOC production is limited and in the absence of photosynthesis, soil processes would be relatively more important than plant processes. Flooding adds an extra dimension to the autumn fluxes, as the water will reduce gas exchanges, create anaerobic environments, dissolve water‐soluble BVOCs, and freeze under more extreme cold conditions, which are all factors that affect BVOC and CO_2_ fluxes. Although autumn BVOC fluxes are considered relatively low, this experiment shows the significant impacts that the expected climate scenarios may have in the future.

## Conflict of Interest

The authors declare no conflicts of interest relevant to this study.

## Supporting information

Supporting Information S1

## Data Availability

The data used in this paper is openly available at ERDA (Electronic Research Data Archive, University of Copenhagen, https://doi.org/10.17894/ucph.77cff72a-5f38-4410-b89f-0eaba4f3347b). Additional figures and tables related to this paper are available in the accompanying Supporting Information S1.
