# Supplementary material for: Bidirectional Exchange of Biogenic Volatile Organic Compounds in Subarctic Heath Mesocosms During Autumn Climate Scenarios
Source: J Geophys Res Biogeosci. 2022 Jun 20;127(6):e2021JG006688. doi: 10.1029/2021JG006688 (PMC9285884; doi:10.1029/2021JG006688)
Supplement: Supplementary file 1 — Supporting Information S1 [file JGRG-127-0-s001.docx]

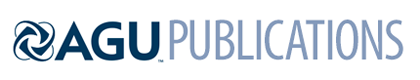


*Journal of Geophysical Research: Biogeosciences*

Supporting Information for

Bidirectional Exchange of Biogenic Volatile Organic Compounds in Subarctic Heath Mesocosms during Autumn Climate Scenarios

Nanna Schrøder Baggesen^1,2^, Cleo L. Davie-Martin^1,2^, Roger Seco^1,2,3^, Thomas Holst^1,4^, Riikka Rinnan^1,2*^.

^1^*Terrestrial Ecology Section, Department of Biology, University of Copenhagen, Universitetsparken 15, DK-2100 Copenhagen Ø, Denmark*

^2^*Center for Permafrost (CENPERM), University of Copenhagen,
Øster Voldgade 10, DK-1350 Copenhagen K, Denmark*

*^3^Institute of Environmental Assessment and Water Research (IDAEA-CSIC),
 Carrer Jordi Girona 18-26, 08034 Barcelona, Catalonia, Spain*

*^4^Department of Physical Geography and Ecosystem Science, Lund University,
Sölvegatan 12, SE-223 62 Lund, Sweden*

**Contents of this file**

Tables S1-S7

Figures S1-S17

**Introduction**

This document includes Tables S1-S7 and Figures S1-S17 that are referenced in the manuscript. Data in the tables and figures were collected simultaneously with data presented in the manuscript and processed alike. Each table and figure has its own caption describing the content.

**Tables**

Table S1. Averaged weather data for October 2002-2021 based on climate data from Abisko Scientific Research Station: https://polar.se/en/research-in-abisko/research-data/. Weather data were used to estimate realistic ambient and cooling_dark autumn weather conditions to use in the experiment.

| October |  |  |  |  | Temperature (°C) | PAR |
| --- | --- | --- | --- | --- | --- | --- |
| Day (8.00-14.00): |  |  |  | min | -15.0 | 0 |
|  |  |  |  | max | 9.5 | 381 |
|  |  |  |  | average | 0.2±0.4 | 188±10 |
| Night (17.00-5.00): |  |  |  | min | -15.2 | 0 |
|  |  |  |  | max | 8.2 | 4 |
|  |  |  |  | average | -1.9±0.4 | 0.1±6.5 |

Table S2. Gravimetric soil water content (%) before flooding (Pre-flooding, 29 March) and after flooding (Flooding, 12 April) (mean ± SE, n = 5). Soil moisture increased significantly after flooding (P < 0.001).

|  |  | Gravimetric soil water content (%) | |
| --- | --- | --- | --- |
| **Climate scenario** |  | Pre-flooding | Flooding |
| Ambient |  | 80.9±0.6 | 87.4±1.5 |
| Cooling_dark |  | 81.1±0.6 | 85.7±0.9 |
| Warming |  | 80.1±0.4 | 87.4±6.4 |

Table S3. Aboveground biomass of the plant species in mesocosms from the ambient, cooling_dark, and warming climate scenarios (g dry biomass m^-2^, mean ± SE, n = 5) for each species and the totals for each plant functional group: graminoids, vascular cryptogams, herbs, deciduous and evergreen shrubs, mosses, lichens, total live biomass, and litter

| Plant species | Ambient | Cooling_dark | Warming |
| --- | --- | --- | --- |
| *Carex* spp. (graminoid) | 12.9±6.3 | 12.0±5.3 | 2.0±1.5 |
| *Equisetum* spp. (vascular cryptogam) | 3.2±1.9 | 1.2±0.7 | 0.8±0.6 |
| *Tofieldia pusilla* | 1.7±1.7 | 1.4±1.1 | 4.8±3.3 |
| *Arctostaphylos alpinus* | - | - | 2.5±2.5 |
| Total herbs | 1.7±1.7 | 1.4±1.1 | 7.3±2.0 |
| *Betula nana* | 25±11 | 62±44 | 41±9 |
| *Vaccinium uliginosum* | 27±14 | 14±5.5 | 25±11 |
| Total deciduous shrubs | 52±8 | 76±22 | 66±7 |
| *Andromeda polifolia* | 31±13 | 24±13 | 8.7±1.8 |
| *Cassiope tetragona* | - | - | 4.1±4.1 |
| *Empetrum hermaphroditum* | 233±70 | 130±54 | 228±110 |
| *Rhododendron lapponicum* | 4.0±4.0 | - | - |
| Total evergreen shrubs | 268±35 | 154±31 | 241±44 |
| Moss | 137±42 | 85±41 | 122±31 |
| Lichen | 5.0±2.2 | 2.4±1.3 | 2.2±2.2 |
| Total live biomass | 480±14 | 332±12 | 441±15 |
| Litter and standing dead biomass | 292±45 | 253±42 | 308±82 |

Table S4. Schematic table of the 20-min program for each of the three freezers. Chamber = the five replicates (1-5) and the blank chamber (blank). Min = duration of each measurement (in minutes) for that particular chamber. Total min = running time (in minutes) for measurements in one freezer.

| Chamber | Min | Total min |
| --- | --- | --- |
| Blank | 1.5 | 1.5 |
| 1 | 2.5 | 4 |
| Blank | 1.5 | 5.5 |
| 2 | 2.5 | 8 |
| Blank | 1.5 | 9.5 |
| 3 | 2.5 | 12 |
| Blank | 1.5 | 13.5 |
| 4 | 2.5 | 16 |
| Blank | 1.5 | 17.5 |
| 5 | 2.5 | 20 |

Table S5. Statistical significance for the factors and interactions affecting BVOC fluxes and NEE during the warmest (hours 15 and 16 – Day) period. P-values from linear mixed effect models are shown for fluxes of methanol, acetic acid, acetaldehyde, acetone, isoprene, monoterpenes, and NEE. Period = the difference between the early and late period within the Pre-flooding and Flooding experiments. Climate scenario = the difference between the three climate scenarios: ambient, cooling_dark, and warming. NA = not included in the final model. Significant values are shown in bold.

| Factor | Methanol | Acetic acid | Acetaldehyde | Acetone | Isoprene | Monoterpenes | NEE |
| --- | --- | --- | --- | --- | --- | --- | --- |
| Period | NA | **<0.001** | 0.961 | **0.014** | **0.001** | NA | 0.054 |
| Flooding | NA | **<0.001** | **0.008** | **0.007** | **0.022** | NA | **0.042** |
| Climate scenario | **0.026** | **<0.001** | 0.114 | **0.045** | 0.124 | NA | **<0.001** |
| Period x flooding | NA | **<0.001** | NA | **0.035** | NA | NA | NA |
| Period x climate scenario | NA | **<0.001** | **0.034** | **0.028** | **0.002** | NA | NA |
| Flooding x climate scenario | NA | **<0.001** | NA | **0.043** | **0.047** | NA | **0.015** |
| Period x flooding x climate scenario | NA | **<0.001** | NA | NA | 0.051 | NA | NA |

Table S6. Statistical significance for the factors and interactions affecting BVOC fluxes and NEE during the coldest (hours 3 and 4 – Night) period. P-values from linear mixed effect models are shown for fluxes of methanol, acetic acid, acetaldehyde, acetone, isoprene, monoterpenes, and NEE. Period = the difference between the early and late period within the Pre-flooding and Flooding experiments. Climate scenario = the difference between the three climate scenarios: ambient, cooling_dark, and warming. NA = not included in the final model. Significant values are shown in bold.

| Factor | Methanol | Acetic acid | Acetaldehyde | Acetone | Isoprene | Monoterpenes | NEE |
| --- | --- | --- | --- | --- | --- | --- | --- |
| Period | NA | 0.565 | 0.722 | NA | **0.009** | NA | NA |
| Flooding | NA | **<0.001** | **<0.001** | **0.039** | **0.036** | NA | 0.115 |
| Climate scenario | **0.042** | 0.569 | 0.276 | 0.146 | **0.012** | NA | **<0.001** |
| Period x flooding | NA | NA | 0.051 | NA | NA | NA | NA |
| Period x climate scenario | NA | NA | **<0.001** | NA | NA | NA | NA |
| Flooding x climate scenario | NA | **0.035** | 0.148 | NA | NA | NA | NA |
| Period x flooding x climate scenario | NA | **0.006** | **0.003** | NA | 0.051 | NA | NA |

Table S7. Greenness (%) of the mesocosms in the three climate scenarios pre-flooding and after flooding (mean ± SE, n = 5). The warming climate scenario's greenness increased significantly (P < 0.005) and the measurement days differed significantly across all climate scenarios (P = 0.013).

|  | Pre- flooding | | |  |  | |  | | |  | After flooding | | |
| --- | --- | --- | --- | --- | --- | --- | --- | --- | --- | --- | --- | --- | --- |
| Climate scenario | 22 March | 26 March | 29 March |  | |  |  |  | 5 April | | | 12 April |  |
| Ambient | 18±1.9 | 16±1.8 | 14±1.3 |  | |  |  |  | 15±1.7 | | | 13±1.5 |  |
| Cooling_dark | 18±1.5 | 14±0.8 | 14±1.5 |  | |  |  |  | 14±0.9 | | | 12±0.9 |  |
| Warming | 7.8±3.7 | 8.6±3.7 | 9.1±3.9 |  | |  |  |  | 9.6±3.1 | | | 12±3.2 |  |

**Figures**

**
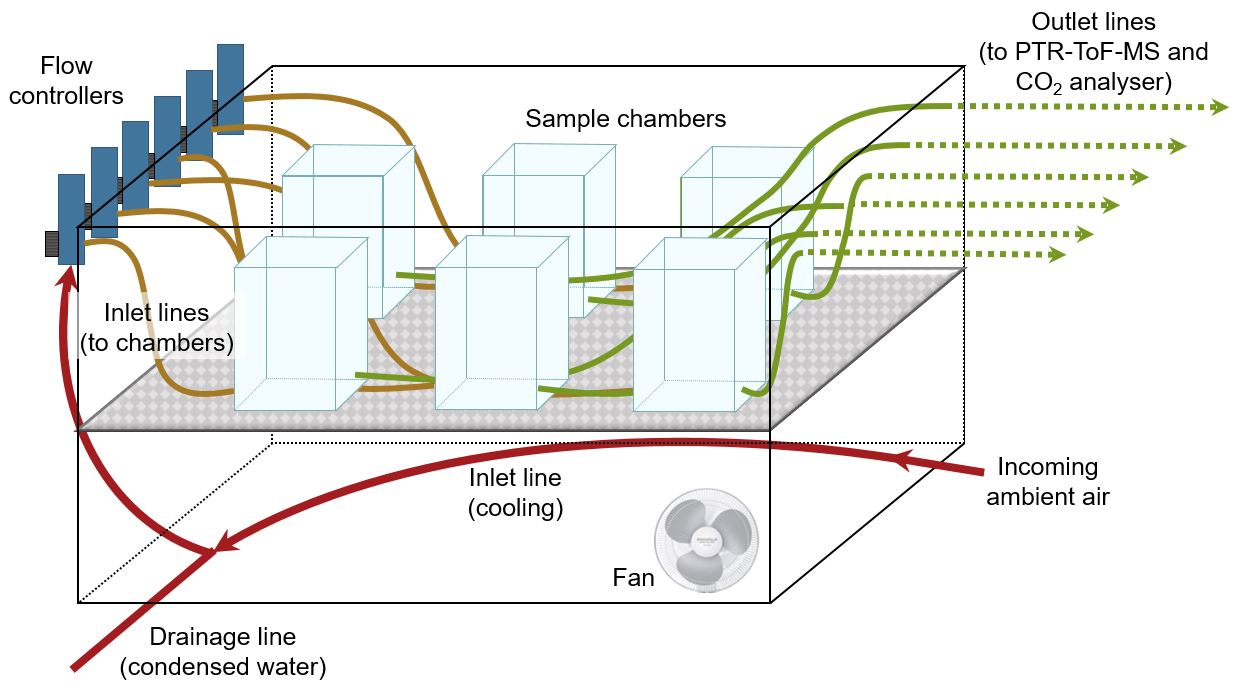
**

Figure S1. Individual climate chamber layout. Filtered ambient air passes through the freezer by way of 3/8” O.D. PFA tubing (dark red lines) to cool the income air stream. A drainage line allows for collection and removal of condensed water. The inlet flow rate to each chamber is set by bubble flow controllers (dark blue with black dial) and enters the polycarbonate sample chambers (light blue) by way of 1/4” O.D. PFA tubing (brown lines). The outgoing sample air (green lines) is transported to the PTR-ToF-MS and CO_2_ analyser by way of 1/4” O.D. PFA tubing. A circulating fan in the base of the climate chamber helps ensure a stable temperature throughout.

**
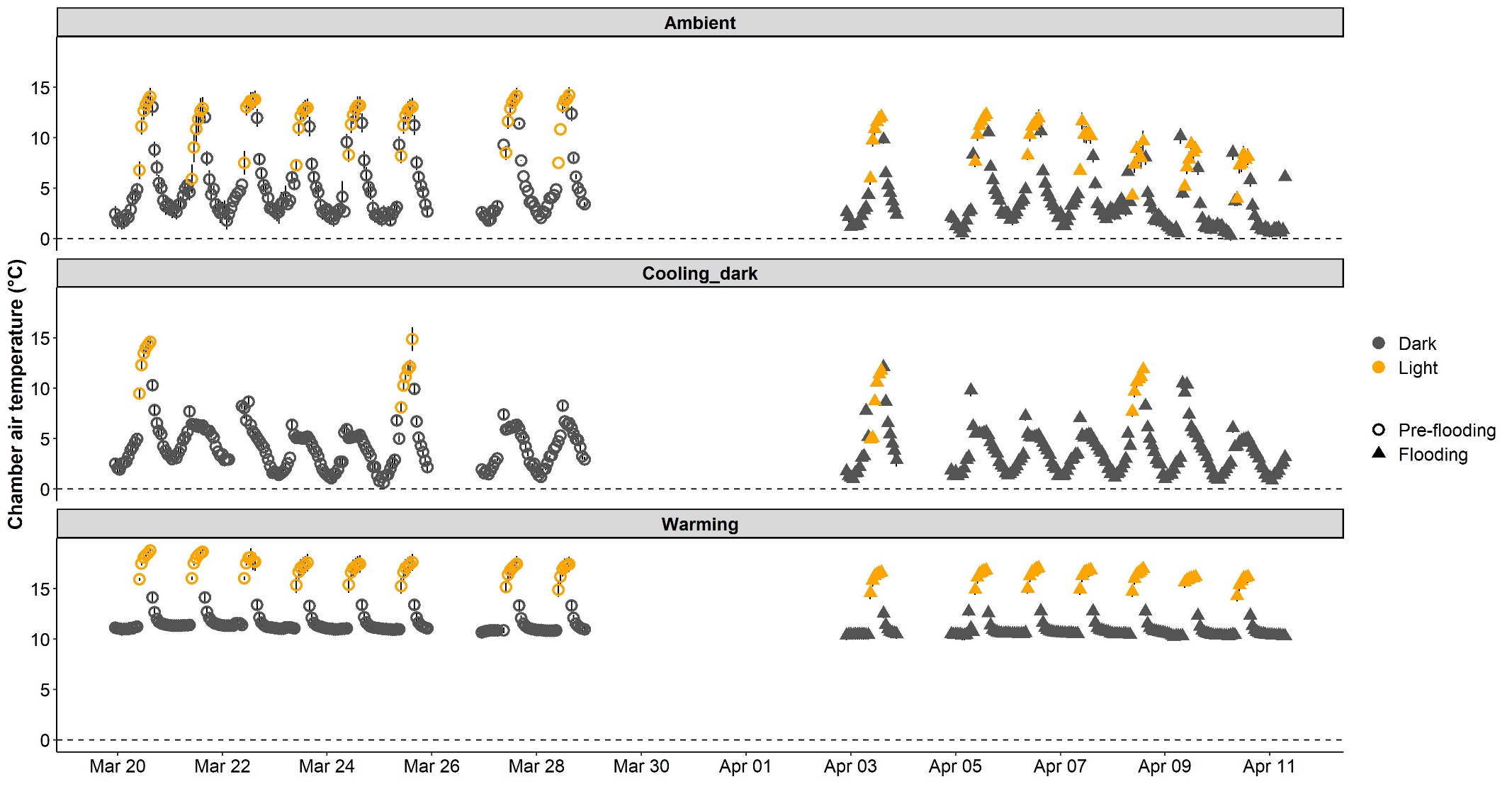
**

Figure S2. Averaged hourly chamber air temperature for each climate scenario (mean ± SE, n = 5) during the experiment.


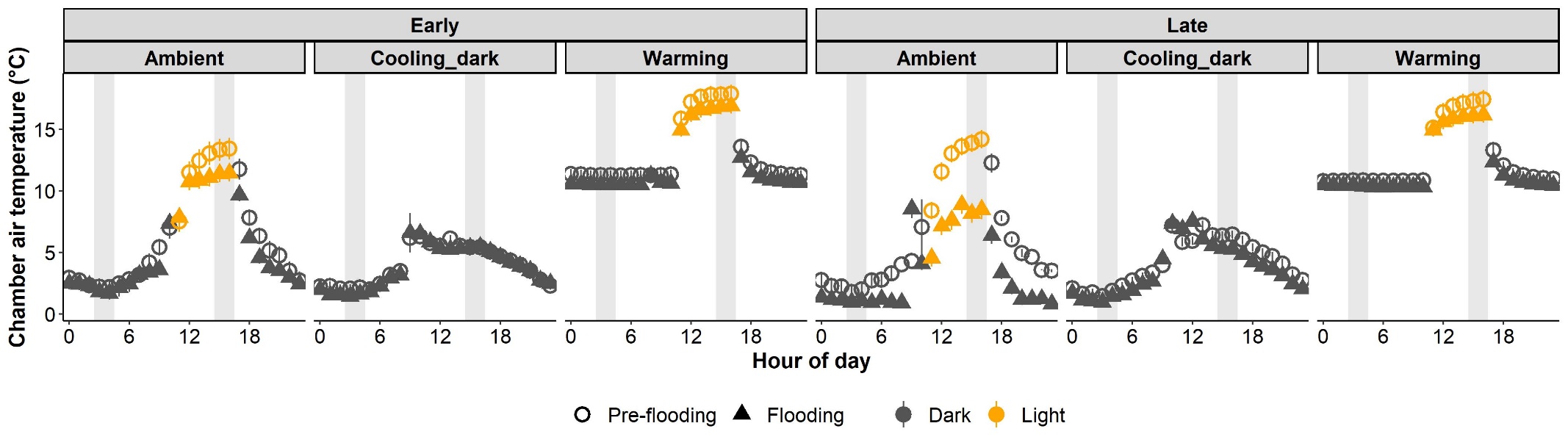
Figure S3. Chamber air temperature for each climate scenario pre-flooding and flooding. Early and late show periods in the beginning and end of each experiment, respectively. Symbols show the mean ± SE, n = 5. Shaded grey bars illustrate data used in the statistical analyses.


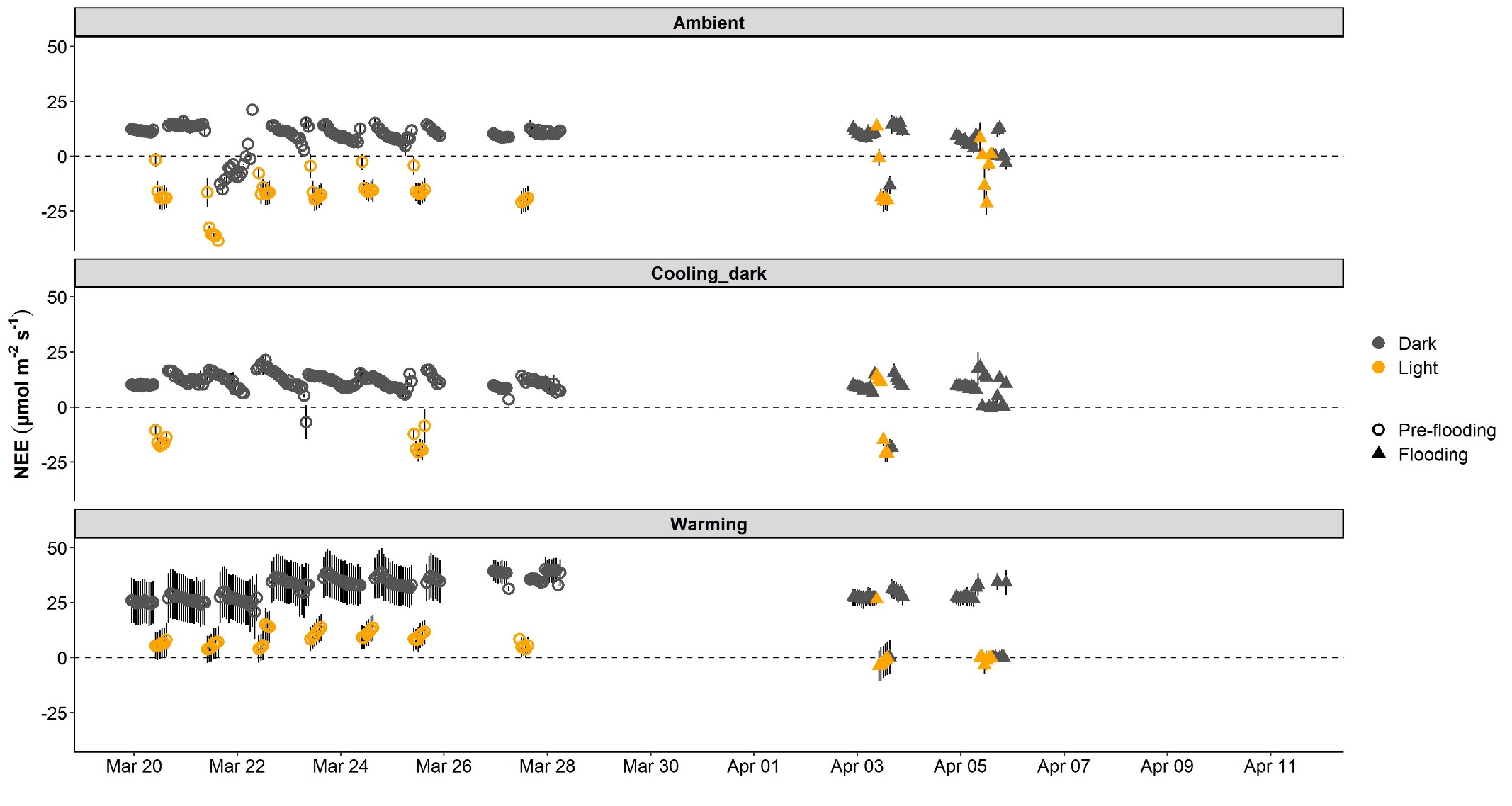


Figure S4. Averaged hourly NEE of CO_2_ for each climate scenario (mean ± SE, n = 5) during the experiments. Positive values depict release from and negative values indicate uptake into the mesocosms.


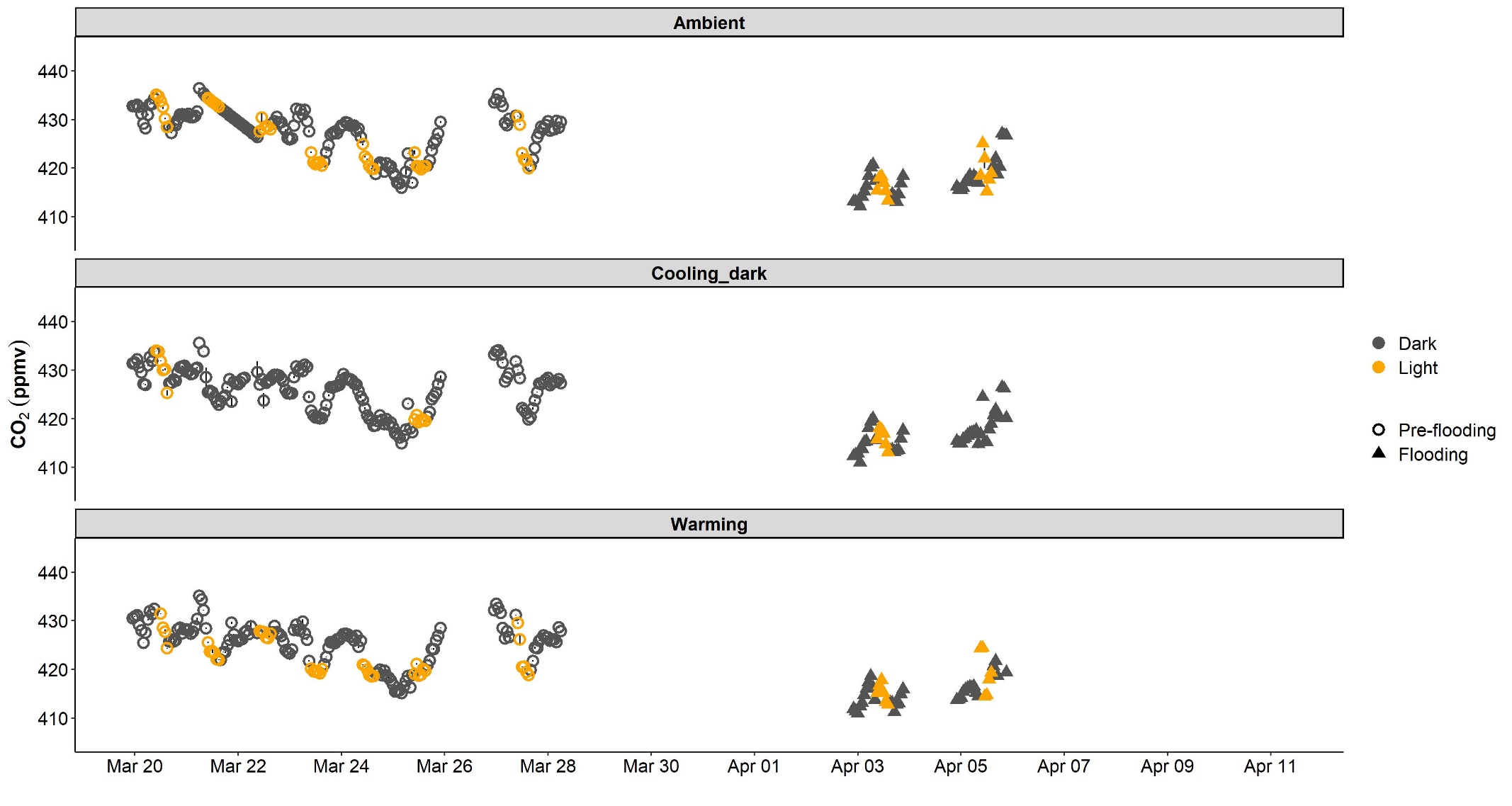


Figure S5. Averaged hourly CO_2_ mixing ratios for incoming background air interpolated for each mesocosm chamber (mean ± SE, n = 5).


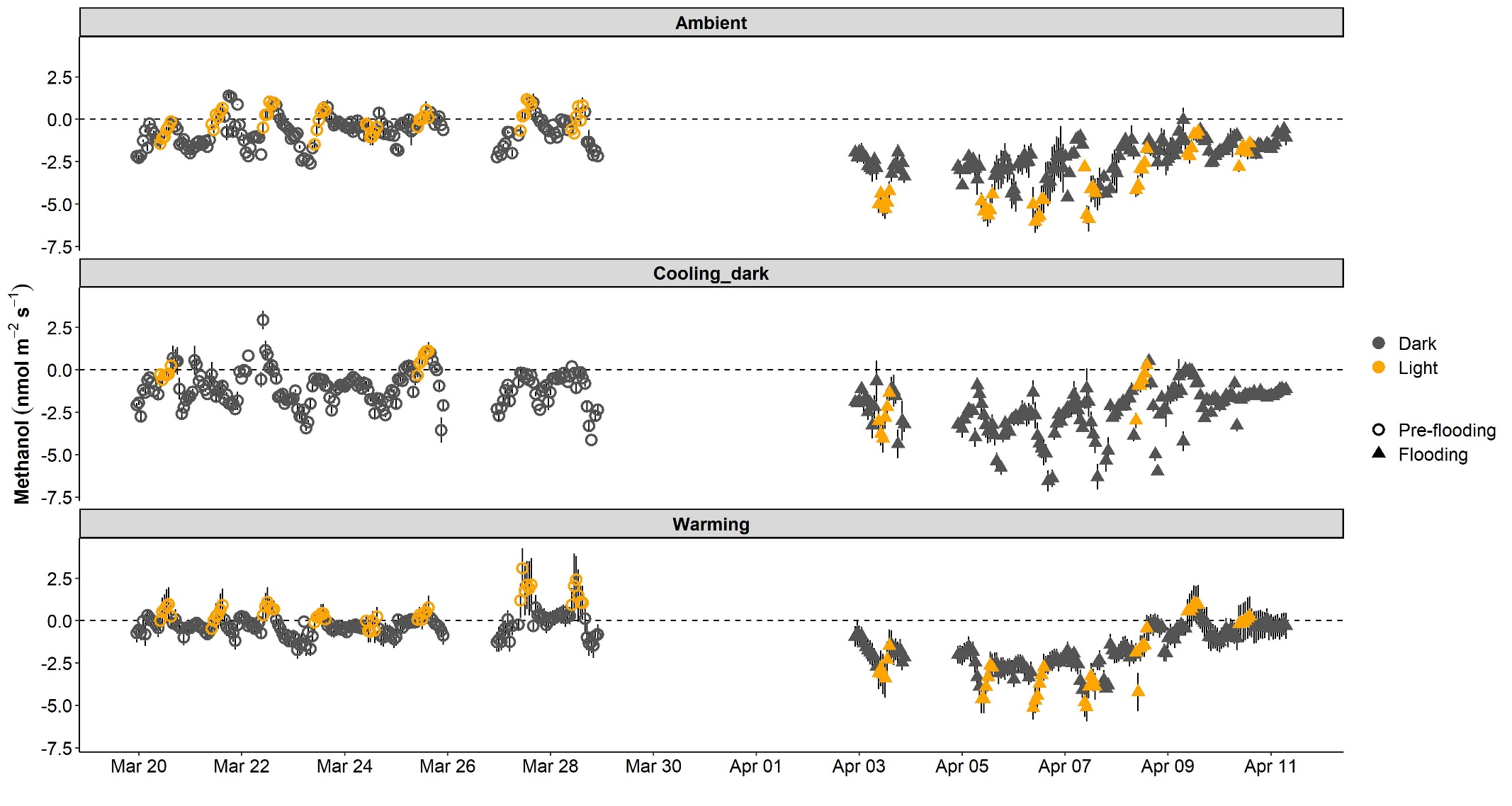


Figure S6. Averaged hourly methanol fluxes for each climate scenario (mean ± SE, n = 5) during the experiments. Positive values depict release from and negative values indicate uptake into the mesocosms.


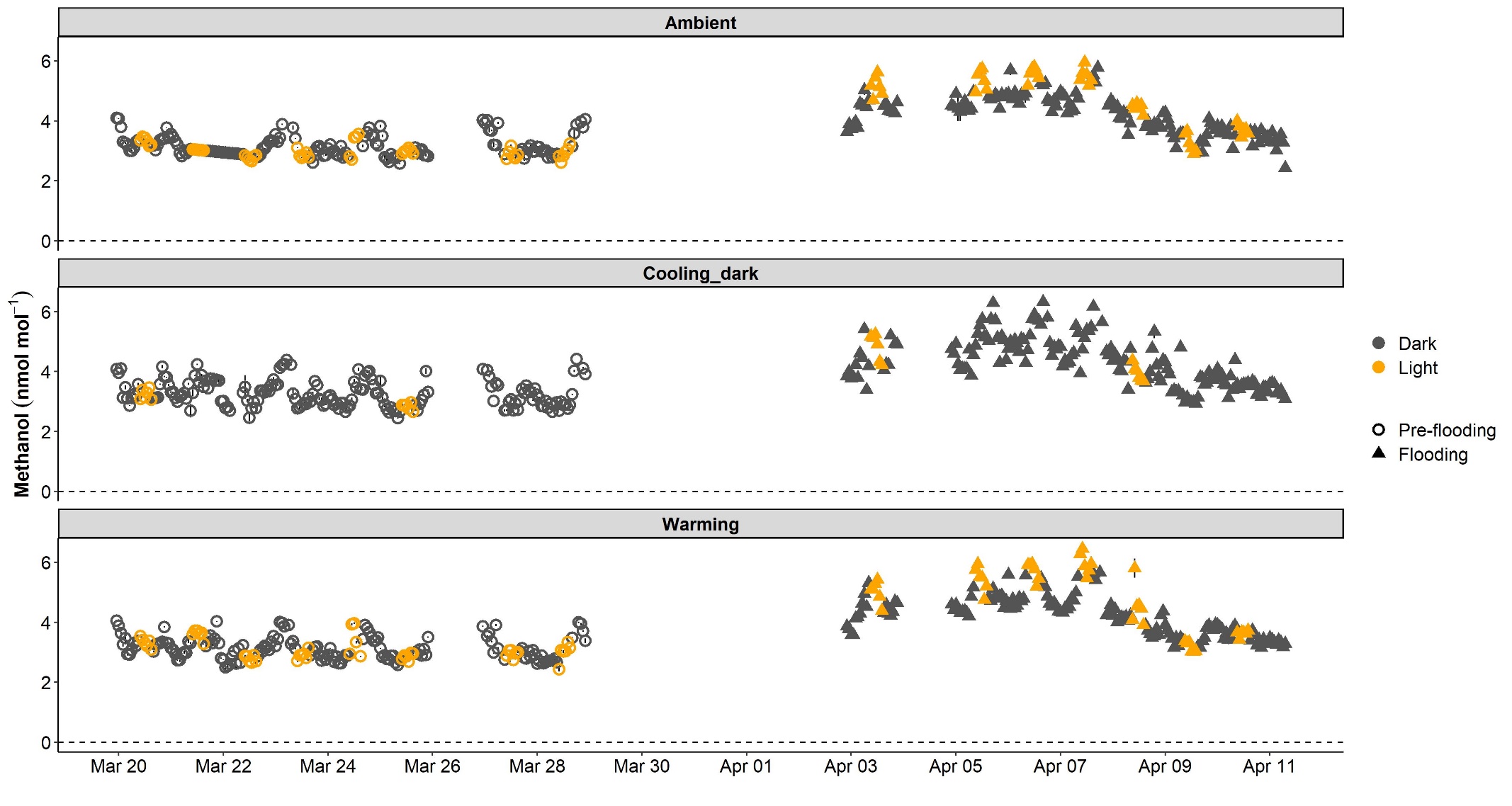
Figure S7. Averaged hourly methanol mixing ratios for incoming background air interpolated for each mesocosm chamber (mean ± SE, n = 5).


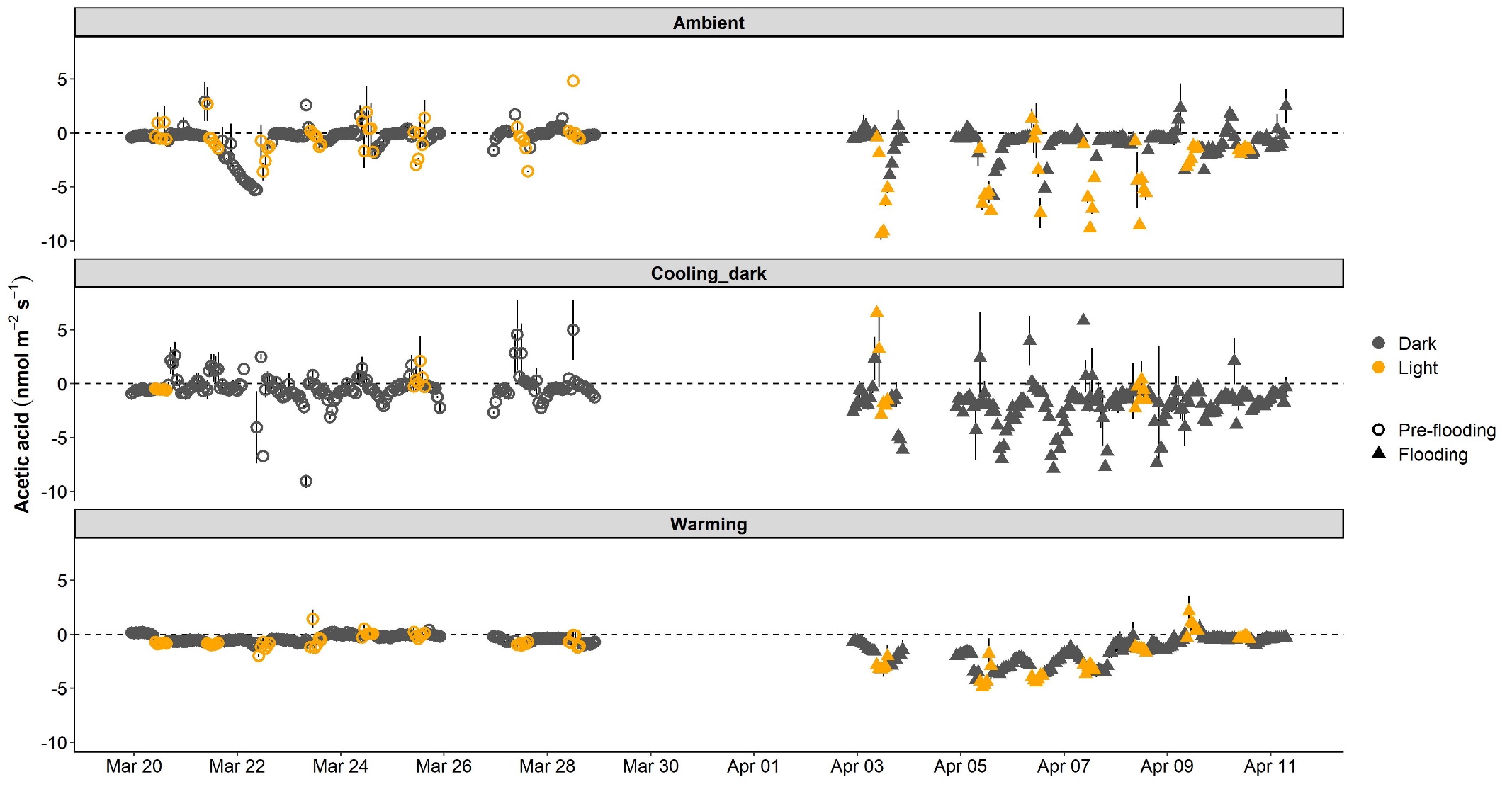
Figure S8. Averaged hourly acetic acid fluxes for each climate scenario (mean ± SE, n = 5) during the experiments. Positive values depict release from and negative values indicate uptake into the mesocosms.


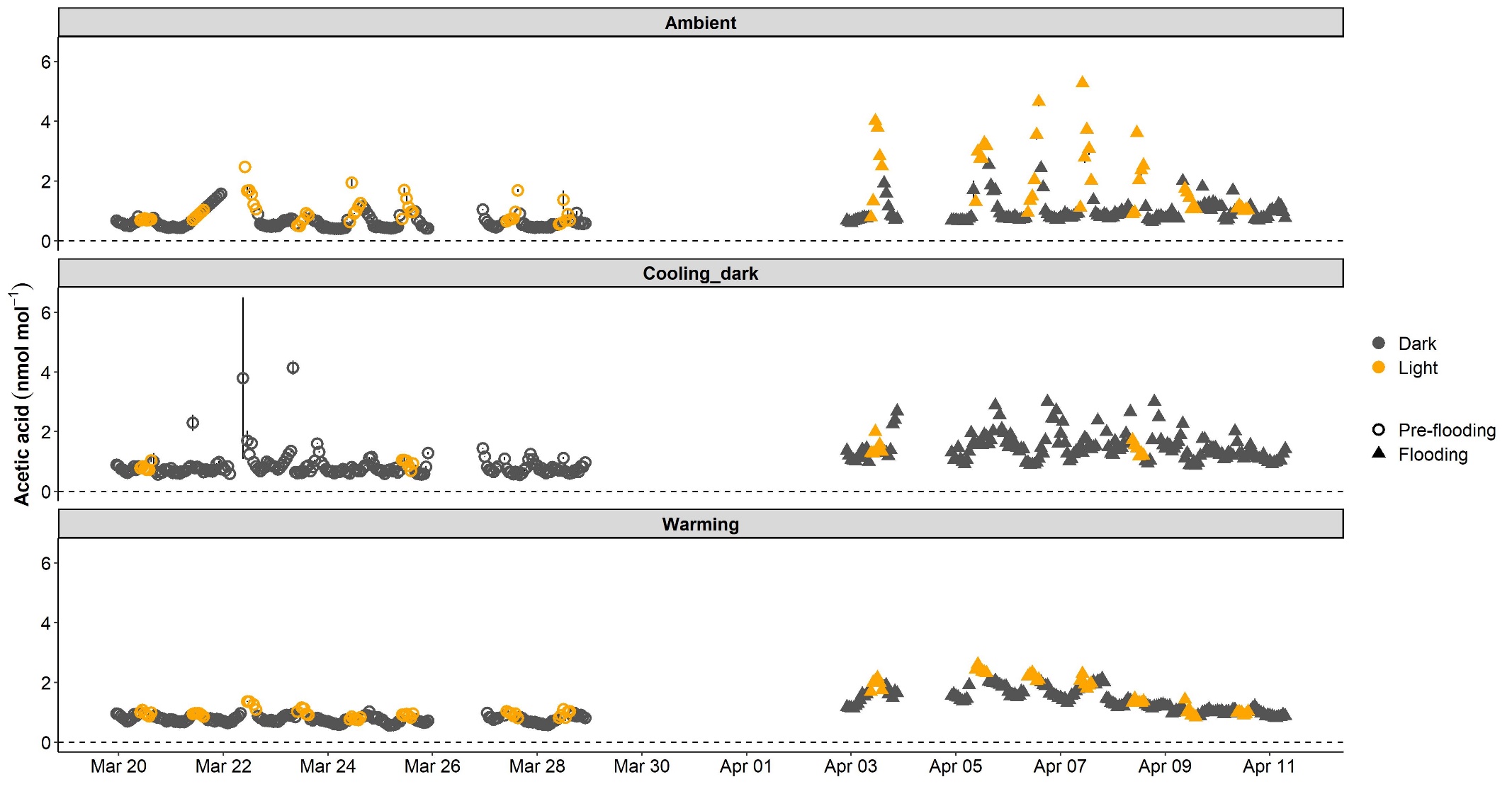
Figure S9. Averaged hourly acetic acid mixing ratios for incoming background air interpolated for each mesocosm chamber (mean ± SE, n = 5).


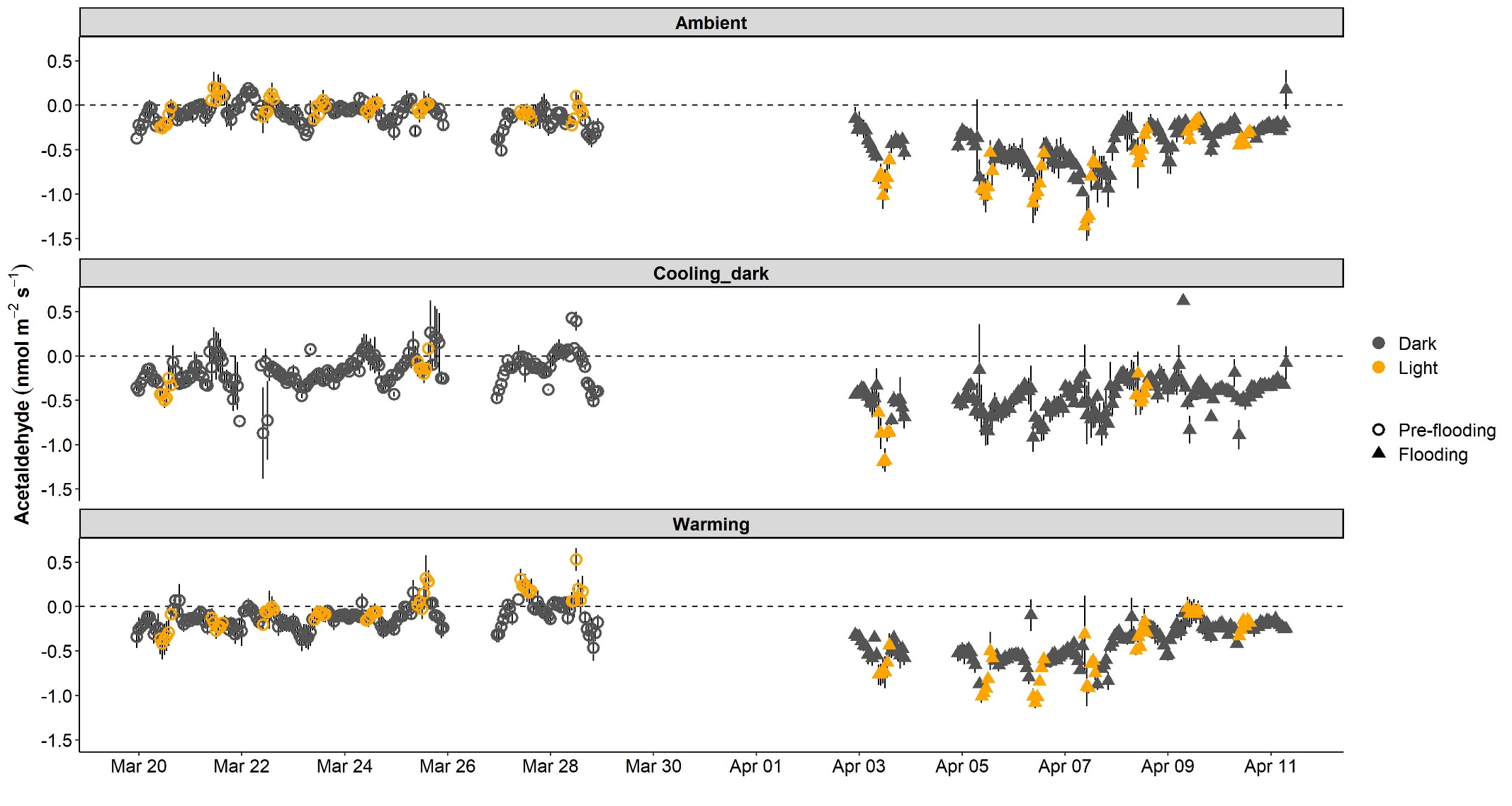
Figure S10. Averaged hourly acetaldehyde fluxes for each climate scenario (mean ± SE, n = 5) during the experiments. Positive values depict release from and negative values indicate uptake into the mesocosms.


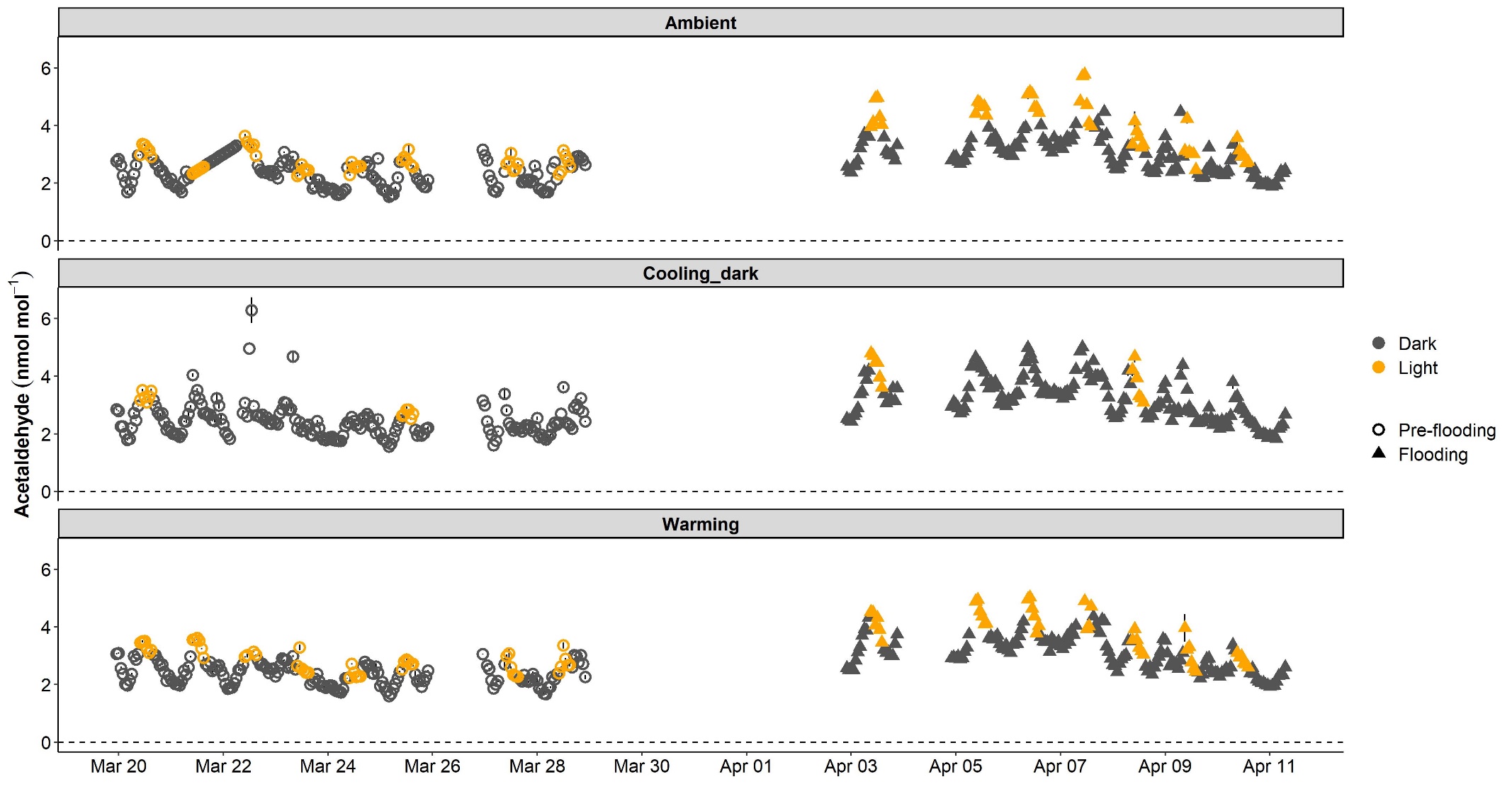
Figure S11. Averaged hourly acetaldehyde mixing ratios for incoming background air interpolated for each mesocosm chamber (mean ± SE, n = 5).


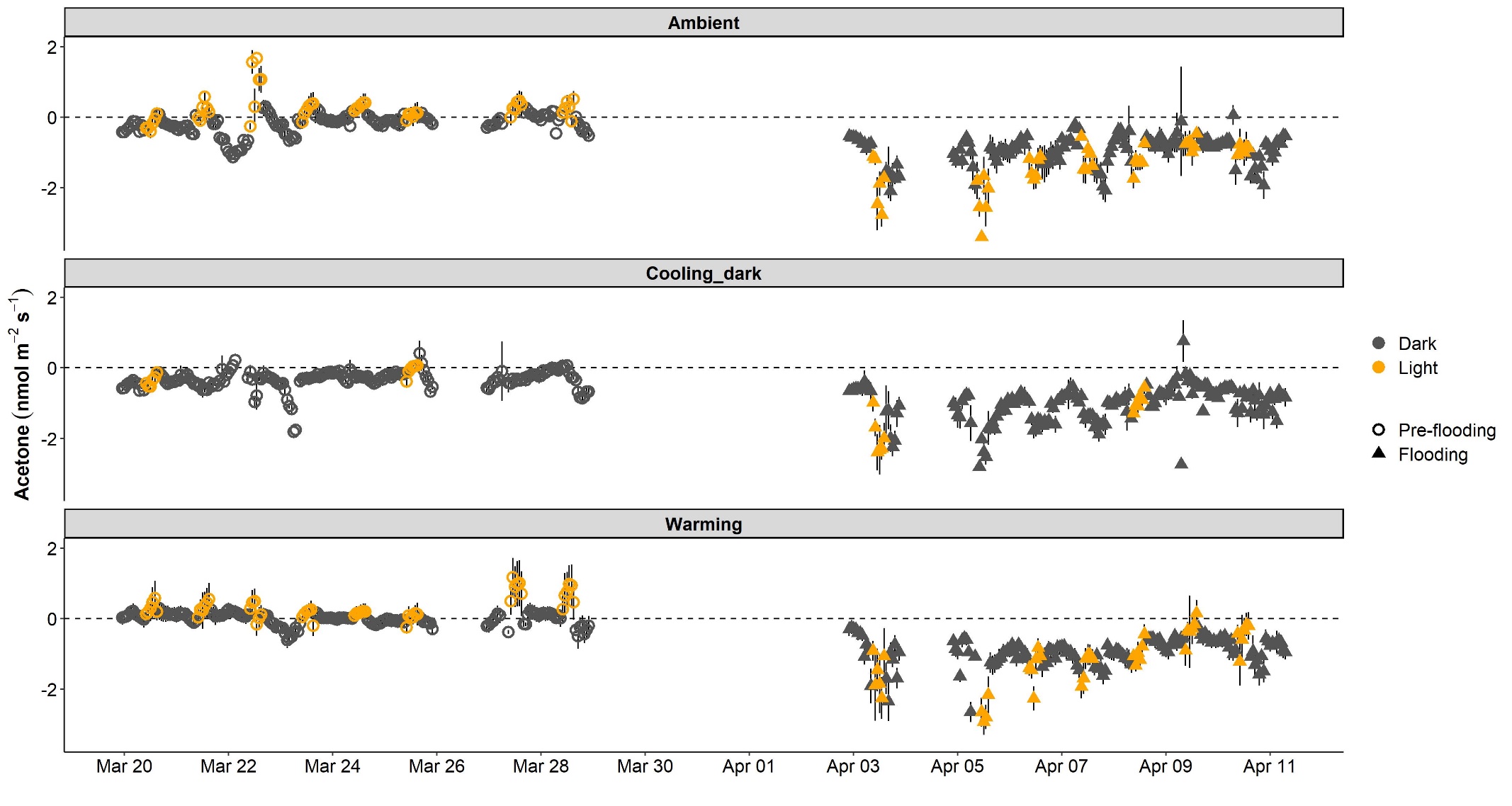


Figure S12. Averaged hourly acetone fluxes for each climate scenario (mean ± SE, n = 5) during the experiments. Positive values depict release from and negative values indicate uptake into the mesocosms.


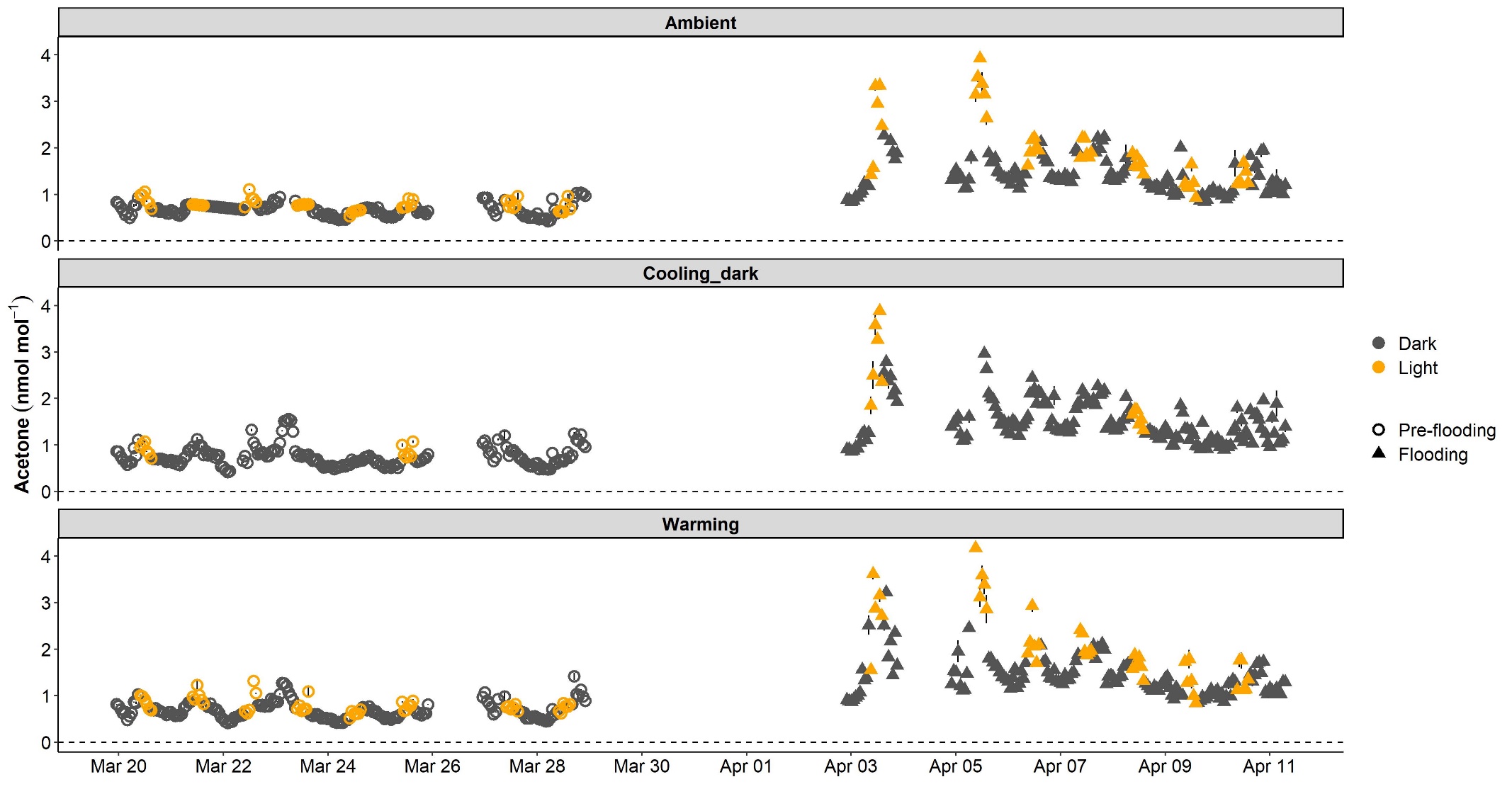
Figure S13. Averaged hourly acetone mixing ratios for incoming background air interpolated for each mesocosm chamber (mean ± SE, n = 5).


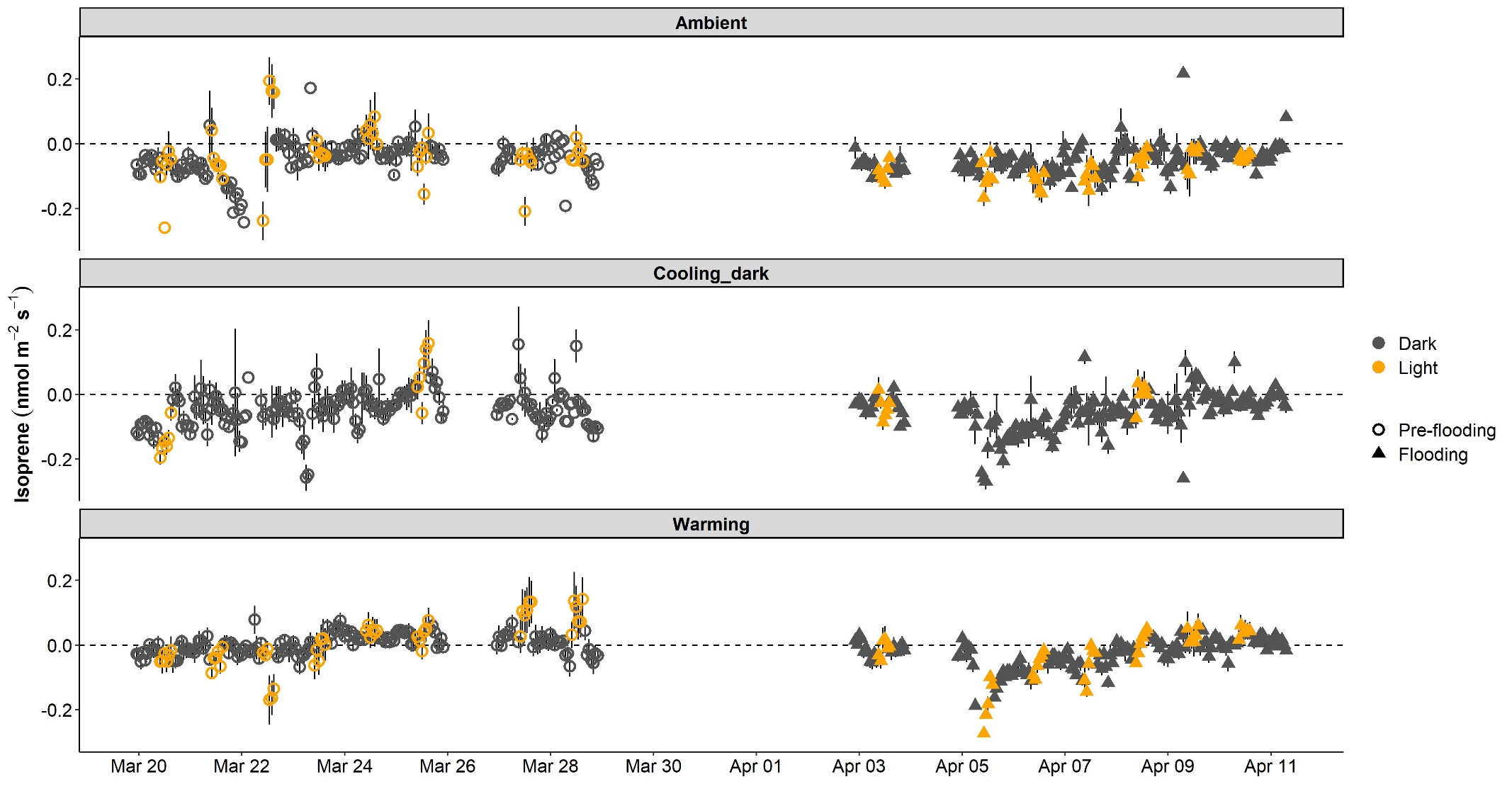


Figure S14. Averaged hourly isoprene fluxes for each climate scenario (mean ± SE, n = 5) during the experiments. Positive values depict release from and negative values indicate uptake into the mesocosms.


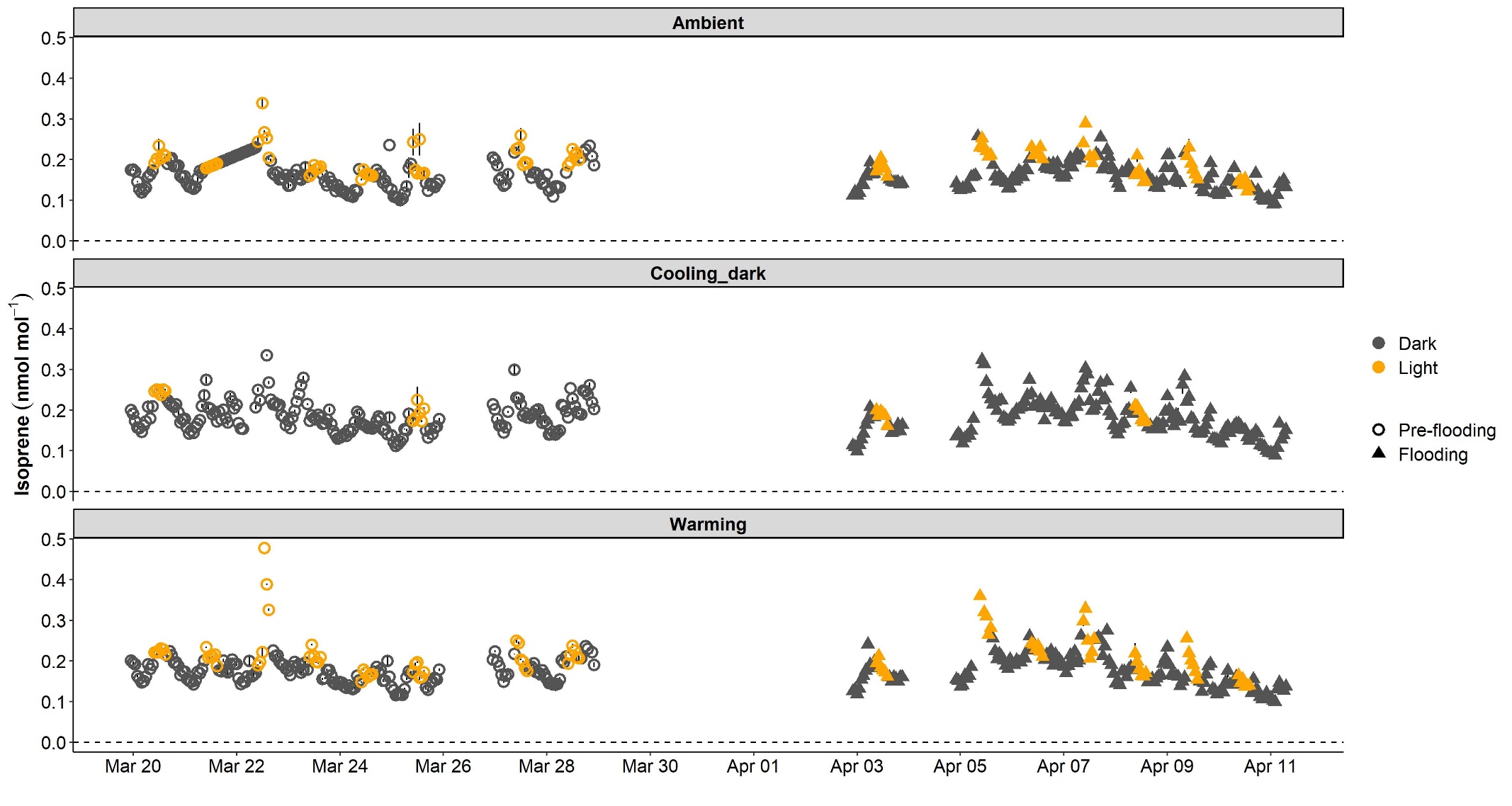


Figure S15. Averaged hourly isoprene mixing ratios for incoming background air interpolated for each mesocosm chamber (mean ± SE, n = 5).


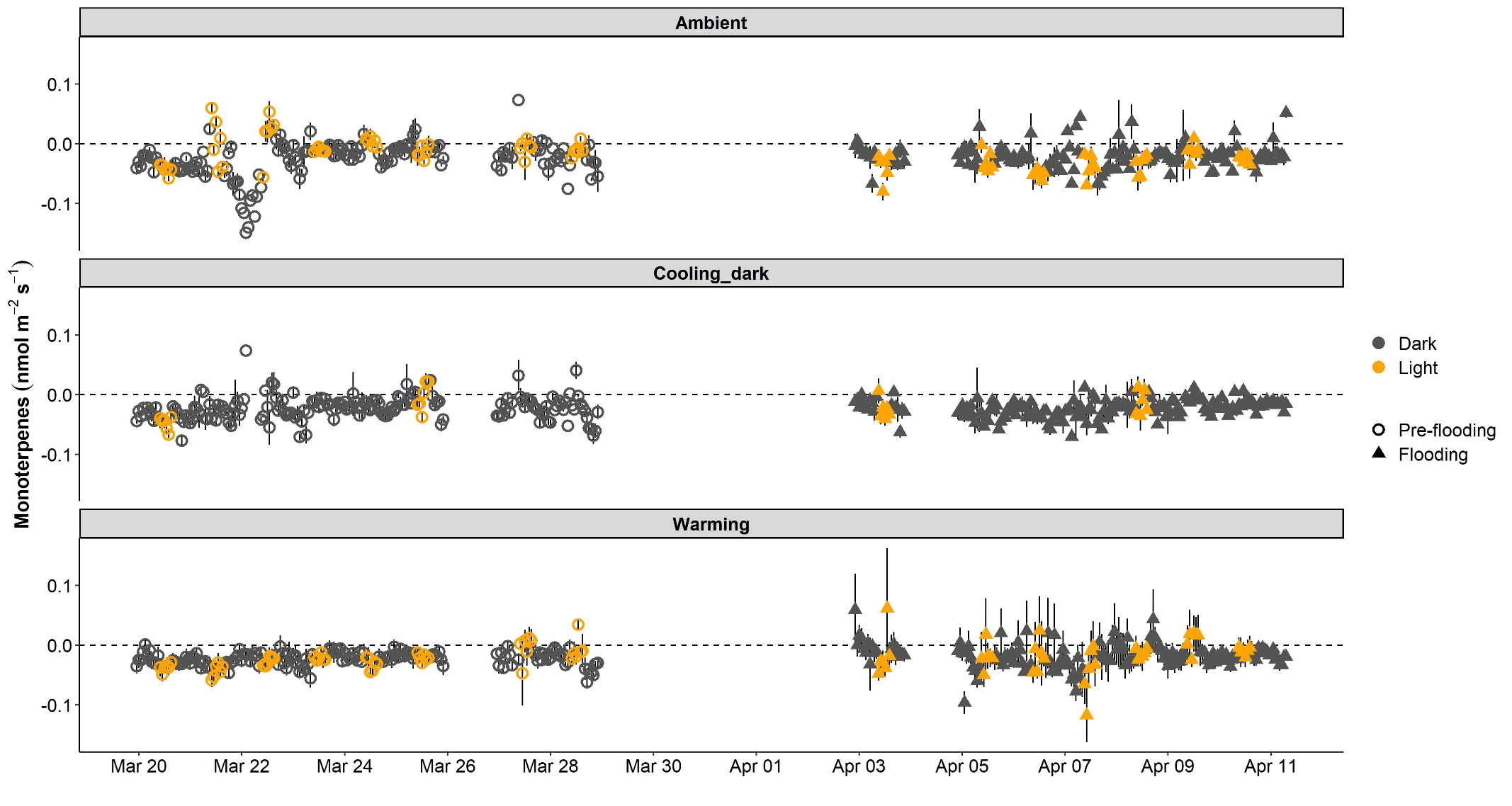


Figure S16. Averaged hourly monoterpene fluxes for each climate scenario (mean ± SE, n = 5) during the experiments. Positive values depict release from and negative values indicate uptake into the mesocosms.


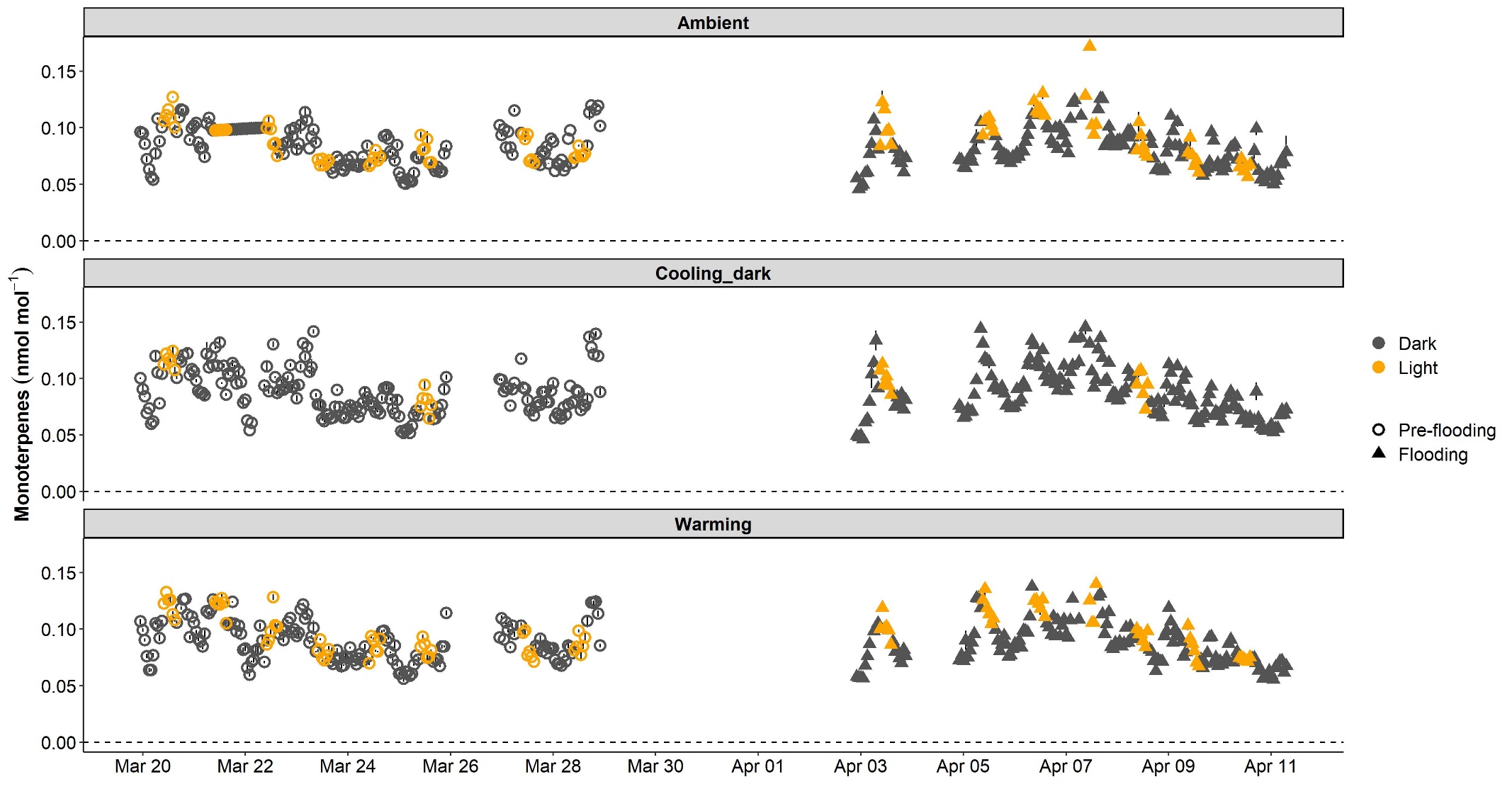


Figure S17. Averaged hourly monoterpene mixing ratios for incoming background air interpolated for each mesocosm chamber (mean ± SE, n = 5).
